# INPP5E controls ciliary localization of phospholipids and the odor response in olfactory sensory neurons

**DOI:** 10.1242/jcs.258364

**Published:** 2021-05-07

**Authors:** Kirill Ukhanov, Cedric Uytingco, Warren Green, Lian Zhang, Stephane Schurmans, Jeffrey R. Martens

**Affiliations:** 1 University of Florida, Department of Pharmacology and Therapeutics, Gainesville, FL 32603, USA; 2 University of Florida, Center for Smell and Taste, FL 32610-0267, USA; 3 Laboratory of Functional Genetics, GIGA-Molecular Biology of Disease, University of Liège, Liège, Belgium

**Keywords:** INPP5E, Mouse, Odor response, Olfactory cilia, Phospholipids

## Abstract

The lipid composition of the primary cilia membrane is emerging as a critical regulator of cilia formation, maintenance and function. Here, we show that conditional deletion of the phosphoinositide 5′-phosphatase gene *Inpp5e*, mutation of which is causative of Joubert syndrome, in terminally developed mouse olfactory sensory neurons (OSNs), leads to a dramatic remodeling of ciliary phospholipids that is accompanied by marked elongation of cilia. Phosphatidylinositol (4,5)-bisphosphate [PI(4,5)P_2_], which is normally restricted to the proximal segment redistributed to the entire length of cilia in *Inpp5e* knockout mice with a reduction in phosphatidylinositol (3,4)-bisphosphate [PI(3,4)P_2_] and elevation of phosphatidylinositol (3,4,5)-trisphosphate [PI(3,4,5)P_3_] in the dendritic knob. The redistribution of phosphoinositides impaired odor adaptation, resulting in less efficient recovery and altered inactivation kinetics of the odor-evoked electrical response and the odor-induced elevation of cytoplasmic Ca^2+^. Gene replacement of *Inpp5e* through adenoviral expression restored the ciliary localization of PI(4,5)P_2_ and odor response kinetics in OSNs. Our findings support the role of phosphoinositides as a modulator of the odor response and in ciliary biology of native multi-ciliated OSNs.

## Abstract (precis)

**Summary:** Cilia of olfactory sensory neurons have a unique lipid composition. Localization of phospholipids is controlled by the INPP5E phosphatase and is involved in modulation of the odor response.

## INTRODUCTION

The olfactory system in animals and humans is optimally tuned to recognize a diverse set of chemical cues and odorants in the environment. In mammals, chemical cues are detected by specialized multi-ciliated olfactory sensory neurons (OSNs) embedded in the olfactory epithelium (OE), transmitting sensory information through action potentials to the olfactory bulb (Firestein, 2001). In most mammalian OSNs, signal transduction is mediated by a canonical cAMP-dependent signaling pathway (Bradley et al., 2005; Kaupp, 2010). Initial binding of an odorant to an olfactory receptor activates a G-protein, Gα_olf_-coupled cascade that triggers the catalytic activity of adenylyl cyclase 3 (AC3, also known as ADCY3) (Brunet et al., 1996; Jones and Reed, 1989) to generate cAMP. When transiently elevated inside cilia, cAMP opens cyclic nucleotide-gated channels (CNGCs) leading to the influx of Ca^2+^ ions, which, in turn, activates the ADCY3-dependent chloride channels (TMEM16B, also known as ANO2) as a secondary amplification cascade (Kaupp and Seifert, 2002; Reisert et al., 2005; Stephan et al., 2009). Importantly, all proteins controlling effective recovery from the transient excitation and overwhelming elevation of intraciliary Ca^2+^, including cAMP hydrolyzing phosphodiesterase 1C, K^+^-dependent Na^+^/Ca^2+^ exchanger and the Ca^2+^ pump, are localized in the ciliary membrane (Cygnar and Zhao, 2009; Mayer et al., 2009; Saidu et al., 2009; Stephan et al., 2012). Despite all the studies that have dissected the main components of this cascade, much less is understood about how the transduction is tuned and regulated within the cilia microenvironment to support optimal sensitivity and resolution of the incoming sensory information.

It is well known that the constituents and composition of the cell membranes act as regulators of signaling proteins that reside in them. Emerging evidence indicates that the lipid composition of cilia may differ from the bulk of the plasma membrane (Lechtreck et al., 2013; Zhao et al., 2012). Surprisingly, until recently very little attention was given to the organization of olfactory cilia, in particular, to the lipid membrane ensheathing the axoneme and harboring both polytopic and peripheral olfactory signaling proteins. A gradually building body of evidence suggests some organizational complexity to the olfactory ciliary bilayer. Our previous work demonstrated a differential partitioning of various lipid-anchored GFP probes that bind to the inner leaflet of the olfactory cilia membrane (Williams et al., 2014). This suggested the presence of ciliary membrane domains with distinct lipid compositions. In addition, the cholesterol-binding protein caveolin-1 (CAV-1) has been implicated as a scaffold to localize proteins in the odor detection pathway to lipid raft domains (Schreiber et al., 2000). In line with these findings, the olfactory CNGA2 channel has been shown not only to have a spatially restricted localization in primary cilium (PC) but also to be functionally regulated by cholesterol (Brady et al., 2004; Jenkins et al., 2006). Another cholesterol binding protein, stomatin-like protein 3 (SLP3; also known as STOML3), was identified in OSNs and localized to the transition zone (TZ) of olfactory cilia (Kobayakawa et al., 2002; Tadenev et al., 2011). Intriguingly, SLP3 co-immunoprecipitated with AC3 and CAV-1 from olfactory cilia isolates (Kobayakawa et al., 2002). Indeed, CAV-1 is not only localized to the PC in other cells types but is also implicated in the regulation of cilia length and sonic hedgehog signaling via a polyphosphoinositide (PI)-dependent pathway (Maerz et al., 2019; Rangel et al., 2019; Schou et al., 2017).

We now know that PIs are involved in specific aspects of sensory function. For example, elevation of phosphatidylinositol (3,4,5)-trisphosphate (PIP_3_) within olfactory cilia can inhibit the CNGCs (Brady et al., 2006; Spehr et al., 2002), whereas odorant stimulation may induce dynamic redistribution of phosphatidylinositol (4,5)-bisphosphate (PIP_2_) in the dendritic knob of OSNs (Ukhanov et al., 2016). Recently, PIs were discovered to play a role in ciliogenesis and regulation of ciliary function (Garcia-Gonzalo et al., 2015; Phua et al., 2017). The interplay between the two PIs, PIP_2_ and phosphatidylinositol 4-phosphate (PI4P), is crucial to the organization of the cilia TZ, and controls protein trafficking and signaling within the PC (Garcia-Gonzalo et al., 2015; Garcia et al., 2018; Phua et al., 2017; Xu et al., 2016). The localization and relative abundance of these two PIs, were found to be in dynamic reciprocity to each other and under the tight control of INPP5E, a phosphoinositide 5′-phosphatase that hydrolyzes PIP_2_ and PIP_3_ (Bielas et al., 2009; Hasegawa et al., 2016; Kisseleva et al., 2000). Each of the PIs, hydrolyzed by INPP5E, represents a small fraction of all membrane-associated lipids, but plays an indispensable role in regulating many aspects of cellular physiology including cell division, vesicle trafficking and control of transmembrane ionic transport (Balla, 2013; Di Paolo and De Camilli, 2006; Hilgemann et al., 2018; Logothetis et al., 2015). Importantly, mutations in the *Inpp5e* gene cause its loss-of-function due to mislocalization or impairment in catalytic activity and manifest in a ciliopathy termed Joubert syndrome (JBTS).

To better understand the role of lipids and specifically PIs in the cell biology of olfactory cilia, we sought to investigate the localization and relative abundance of several lipids by utilizing a conditional *Inpp5e*-deficient mouse mutant. A panel of highly selective probes to several important classes of lipids were used in live mouse OSNs *in situ*. Using this approach and mouse model allowed us, for the first time, to analyze the distribution and functional implication of JBTS ciliopathy-related changes to the phospholipid composition of cilia in terminally differentiated mammalian sensory neurons.

## RESULTS

### Loss of *Inpp5e* causes PIP_2_ redistribution from the knob into the olfactory cilia

**Fig. 1.**
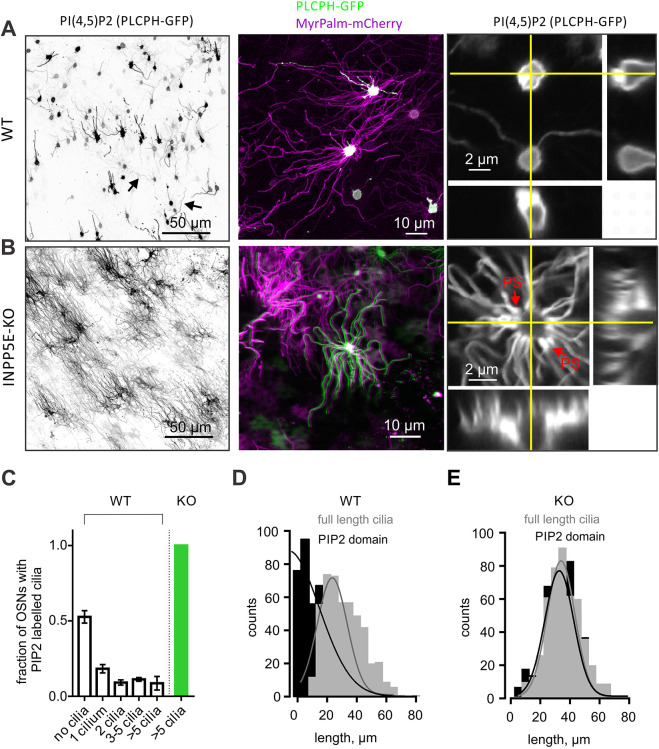
**Loss of INPP5E causes redistribution of PIP_2_ and elongation of cilia in mouse OSNs.** (A) PLCPH–GFP, a probe for PIP_2_, is mostly restricted to the knob of WT OSNs. In a small percentage of OSNs, a ciliary segment of varying length up to the full length (black arrows) is also enriched in PIP_2_. The inert membrane-bound lipid probe MP–mCherry was used as a counterstain to label the full length of axoneme, and does not have the highly restricted localization of PLCPH-GFP resulting in overlapping colors (middle panel, white). PIP_2_ was evenly distributed in the plasma membrane of the knob as shown in *z*-stack view (right panel). Yellow lines denote *z*-stack projection shown at the bottom and right side of the image. (B) In contrast to what is seen in the WT, in *Inpp5e*^osnKO^ (INPP5E-KO) PLCPH–GFP decorated the entire length of every cilium. A color-shifted image is shown to accentuate the equal distribution of PLCPH–GFP and MP–mCherry labeling (B, middle panel). The PIP_2_ redistribution is evident also in the *z*-stack side view showing substantial enrichment at the base of cilia and along the proximal segment (PS, red arrows) whereby PIP_2_ level in the knob periciliary plasma membrane was not changed (right panel). (C) More than 50% of WT OSNs showed no PIP_2_ in their cilia. 18% of OSNs had only a single PIP_2_-positive cilium whereas three other groups of neurons equally represented the remaining 30%. Conversely, PIP_2_ was detected in 100% of OSNs in *Inpp5e*^osnKO^ (KO, green bar). A total of 318 cells in 4 mice were analyzed in the WT group and 36 cells in 3 mice were analyzed in the KO group. (D) Length distribution within the same sets of cells of PLCPH–GFP-positive aspects of cilia (PIP_2_ domain) in WT was substantially shifted to shorter values compared to the full cilia length measured with MP-mCherry, yielding 29.5±0.5 µm (*n*=753, 4 mice). (E) Distribution of both PLCPH–GFP and MP–mCherry length values showed a complete overlap in *Inpp5e*^osnKO^ OSNs. The average full ciliary length in the KO OSNs, 35.3±0.6 µm (*n*=495, 3 mice) was significantly longer than in the WT (unpaired *t*-test, t=7.363, d.f.=1246, *P*<0.0001). Data shown as mean±s.e.m.

INPP5E hydrolyzes two phosphoinositide species PIP_2_ and PIP_3_ with high affinity generating PI4P and phosphatidylinositol (3,4)-bisphosphate [PI(3,4)P_2_], respectively (Kisseleva et al., 2000; Kong et al., 2000). Distribution of PIP_2_ in mature OSNs was measured using *en face* confocal microscopy of intact olfactory epithelium transduced with adenovirus encoding the PLCδ1-PH (PLCPH) domain tagged with GFP (Fig. 1). In wild-type (WT) littermate control mice, 52.7±10.8% (*n*=318, 4 mice) of cells infected with the PLCPH probe had an extremely polarized distribution of PIP_2_ with an accumulation in the OSN knob and no ciliary localization (Fig. 1A,C; Fig. S1B; note, all results presented in the main text are given as mean±s.e.m.). In those cells, PIP_2_ was uniformly distributed in the plasma membrane of the knob and adjacent dendrite and extended all the way to the axons (Fig. 1A, right panel; Fig. S1B,C). The total number of cilia (21.8±0.5 cilia per OSN; *n*=37, 4 mice) and cilia length (29.5±0.5 µm; *n*=753, 4 mice) was measured by co-expression of an inert lipid-anchored probe MyrPalm fused to mCherry (MP-mCherry) (Fig. 1A, middle panel). In a fraction of OSNs, however, we detected PIP_2_ in a small subset of cilia ranging from one to five cilia per neuron, but this allocation was much fewer than the total number of cilia (Fig. 1C; Fig. S1B). The distribution of PIP_2_ along the length of a given cilium was highly variable and ranged from a short segment to the full length (Fig. 1A,D). A full-length distribution of PIP_2_ was rare and often seen in only a single cilium. Overall distribution of PIP_2_ and MP-mCherry in the WT cilia resulted in non-overlapping histograms, as summarized in Fig. 1D.

To get insight in the regulation of phospholipids, in particular PIP_2_, in olfactory cilia and OSNs, we utilized an olfactory-specific conditional knockout mouse *Inpp5e*^osnKO^. The mutant was generated by crossing *Inpp5e*^loxP^ founder described previously (Jacoby et al., 2009) with a mouse carrying Cre-recombinase under the promoter of the olfactory marker protein (OMP), which is expressed exclusively in mature OSNs (Green et al., 2018). Consistent with previous transcriptomic and proteomic data in OSNs (Kuhlmann et al., 2014; Nickell et al., 2012), western blot data (representative images and densitometry) of OE extracts show protein expression of a doublet at ∼72 kDa, corresponding to the WT INPP5E and a splice variant (Jacoby et al., 2009), that is decreased in the *Inpp5e*^osnKO^ mouse (Fig. S1A). The remaining signal likely reflects the presences of multiple cell types in the OE. The loss of INPP5E in OSNs of *Inpp5e*^osnKO^ mouse severely impacted ciliary PIP_2_ distribution resulting in its homogenous redistribution along the entire axoneme (Fig. 1B). Remarkably, this deficiency affected every cilium (Fig. 1C, KO) shifting distribution of PIP_2_ domain length to a complete overlap with that of the ciliary length marker MP-mCherry (Fig. 1E). Another salient feature of the PIP_2_ localization in the KO cilia was its abundance within the proximal segment of each cilium, overlapping with the TZ (Fig. 1B, right panel, red arrows). Notably, the mean cilia length was significantly increased from 29.5±0.5 µm in WT littermates to 35.3±0.6 µm in the *Inpp5e*^osnKO^ mice (*n*=495, 3 mice, unpaired *t*-test, t=7.363, d.f.=1246, *P*<0.0001). Cilia length is controlled by an evolutionarily conserved process of intraflagellar transport (IFT) (Rosenbaum and Witman, 2002). The loss of INPP5E impacts IFT in primary cilia, resulting in the selective accrual of IFT-A particles (Chávez et al., 2015; Garcia-Gonzalo et al., 2015). Surprisingly, we did not find any abnormality in the velocity of IFT-A-dependent transport of IFT122 particles or its accumulation inside olfactory cilia of *Inpp5e*^osnKO^ mice (Fig. S2A,B, Movie 1). IFT-B-related trafficking of IFT88 was also unaltered, with a similar particle velocity to that published previously for the wild-type OSNs (Uytingco et al., 2019; Williams et al., 2014) (Fig. S2C–E, Movie 2).

### Ectopic expression of human *INPP5E* restores the restricted distribution of PIP_2_ in *Inpp5e*^osnKO^ OSNs

**Fig. 2.**
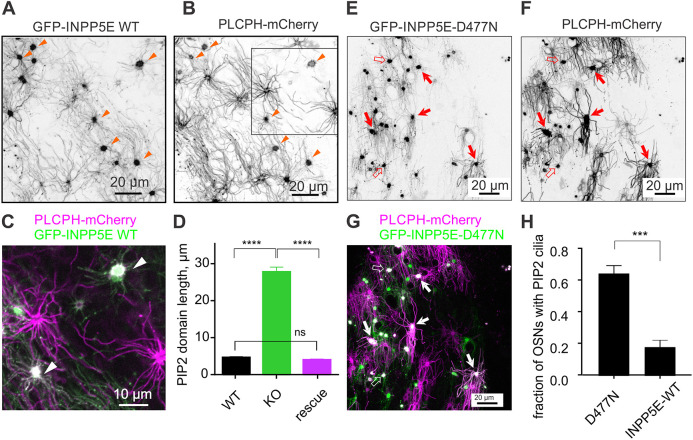
**Virally induced ectopic expression of full-length WT human INPP5E tagged with GFP completely** **reversed mislocalization of PIP_2_ in *Inpp5e*^osnKO^ mouse cilia.** (A,B) *Inpp5e*^osnKO^ mice were infected at P8–P14 with a triple dose of Ad-GFP-INPP5E-WT and tested 8–10 days later. GFP–INPP5E-WT is enriched in OSN knobs and also localizes to cilia. The KO mice were co-infected with PLCPH–mCherry to measure rescue of the PIP_2_ localization. Several knobs of co-infected OSNs are indicated with arrowheads. (C) Magnified dual-color view of the area marked with a square in B shows several knobs of OSNs co-infected with both viruses (arrowheads) resulting in a complete loss of ciliary PIP_2_ (magenta). (D) Rescue was quantified by measuring length of PIP_2_ positive ciliary aspect in the WT littermates and KO mice. The KO OSNs were identified within the same preparation by a strong ciliary distribution of PLCPH–mCherry, and also lacking any detectable GFP–INPP5E-WT fluorescence. Rescue completely reversed *Inpp5e*^osnKO^ deficiency [PIP2 domain length 4.9±0.27 µm (*n*=110, 16 cells, 3 mice), WT; 28.5±1.37 µm (*n*=54, 5 cells, 3 mice), KO; 4.2±0.3 µm (*n*=122, 17 cells, 3 mice), Rescue, one-way ANOVA, F(DFn, DFd) 86.73 (2283), ****P<0.0001]. ns, not significant. (E,G) *Inpp5e*^osnKO^ KO mice in a different group were infected with Ad-PLCPH-mCherry and Ad-GFP-INPP5E-D477N encoding for catalytically inactive phosphatase. The GFP–NPP5E-D477N mutant was localized to the full cilia length (E). Knobs of co-infected OSNs showing no change in PLCPH ciliary localization are marked with solid arrows. Some knobs had less PLCPH probe localized to cilia (open arrows) reminiscent of the KO phenotype. (G,H) Expression of GFP–INPP5E-D477N resulted in a significantly smaller number of OSNs having a complement of PIP2-decorated cilia. This reduction was quantified in H, 17.6±0.09% (D477N, *n*=61, 3 mice), compared to GFP–INPP5E-WT, 61.2±0.05% (INPP5E-WT, *n*=83 cells, 3 mice), unpaired *t*-test, t=4.536, d.f.=24, ***P=0.001). Data shown as mean±s.e.m.

To address the potential of virally assisted therapy of the JBTS ciliopathy model *in vivo*, we used a rescue adenoviral vector carrying the full-length sequence of human *INPP5E* (NM_019892) fused with GFP on the N-terminal, GFP–INPP5E-FL (Chávez et al., 2015). Ectopically expressed GFP–INPP5E-FL was enriched in the OSN knobs and localized to the full length of cilia in the WT (Fig. S1D) and KO mouse (Fig. 2A). As shown in Fig. 2, full-length WT INPP5E was necessary for restoration of normal PIP_2_ distribution in OSNs. Ectopic expression of GFP–INPP5E-FL in *Inpp5e*^osnKO^ OSNs resulted in a significant decrease of PIP_2_ ciliary domain length as measured with PLCPH-mCherry (Fig. 2B,C). The average length of the PIP_2_ domain in WT cilia was 4.9±0.27 µm (*n*=110, 16 cells, 3 mice), in *Inpp5e*^osnKO^ cilia 28.5±1.37 µm (*n*=54, 5 cells, 3 mice) and in rescued KO cilia 4.2±0.3 µm (*n*=122, 17 cells, 3 mice) [*P*<0.0001, one-way ANOVA, F(DFn, DFd) 86.73 (2283)] (Fig. 2D). As a negative control we used a catalytically inactive point mutant GFP–INPP5E-D477N (Chávez et al., 2015), which failed to change localization of PIP_2_ when co-expressed with PLCPH–mCherry in HEK293 cells (Fig. S3). Co-expression of PLCPH–mCherry with GFP–INPP5E-D477N resulted in a significantly larger number of OSNs having a complement of PIP_2_-decorated cilia, 61.2±0.05% (D477N, *n*=61, 3 mice) compared to INPP5E-WT, 17.6±0.09% (INPP5E-WT, *n*=83, 3 mice) (*P*=0.0001, unpaired *t*-test, t=4.536, d.f.=24) (Fig. 2E–H). Together, these data indicate that the catalytic activity of INPP5E is required for restricting the distribution of PIP_2_ in olfactory cilia.

### Loss of *Inpp5e* affects multiple phospholipids in OSNs

**Fig. 3.**
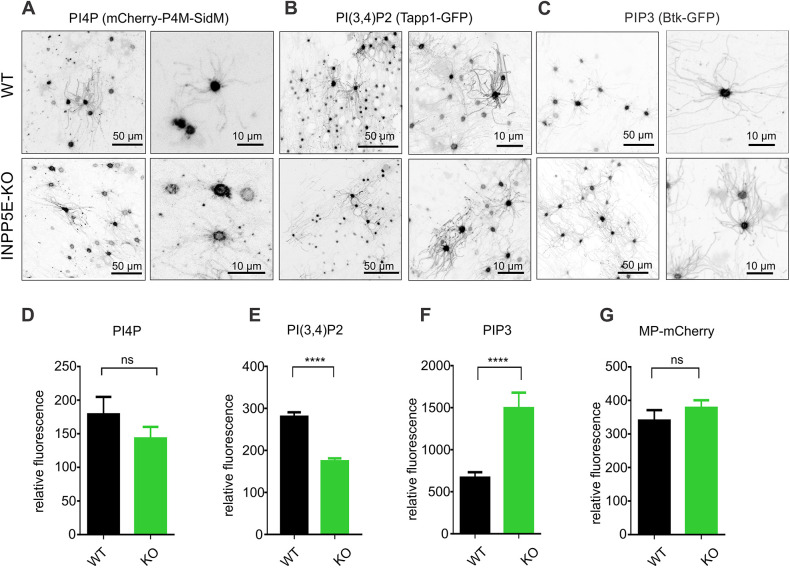
**Other phosphoinositides than PIP_2_** **in mouse OSNs are almost exclusively restricted to the knobs and changed their level in an INPP5E-dependent manner.** (A,D) The location of the PI(4)P probe mCherry–P4M-SidM was not significantly affected by loss of INPP5E showing only insignificant trending decrease in the knobs (179±26 relative units, WT, *n*=94, 3 mice; 143±17 relative units, KO, *n*=54, 3 mice; unpaired *t*-test, t=0.9777, d.f.=146, *P*=0.3298). (B,E) A tandem PH domain, Tapp1 tagged with GFP, was used to specifically label membrane PI(3,4)P_2_, which was found to only be enriched in the knobs and in cilia in a small fraction of OSNs. Importantly, the overall pattern of PI(3,4)P_2_ distribution did not change in *Inpp5e*^osnKO^. Fluorescence intensity, however, measured in OSN knobs showed a significant decrease in the KO compared to WT mice (280±11 relative units, *n*=830, 3 mice, WT; 174±7 relative units, *n*=858, 3 mice, KO; unpaired *t*-test, t=8.453, d.f.=1686, *P*<0.0001). (C,F) PIP_3_ detected with a Btk-PH domain tagged with GFP, was restricted mostly to the knobs with a relatively low presence in cilia of the WT and KO. Quantitative analysis of fluorescence showed increase of the intensity in the knobs of the KO (668±64 relative units, *n*=60, 3 mice, WT; 1495±185 relative units, *n*=91, 3 mice, KO; unpaired *t*-test, t=3.536, d.f.=149, ***P=0.0005). (G) Fluorescence intensity of MP–mCherry, used as a negative control, was not significantly different in the OSN knobs of WT and KO mice (340±31 relative units, *n*=46, 3 mice, WT; 378±23 relative units, *n*=70, 3 mice, KO; unpaired *t*-test, t=1.001, d.f.=114, *P*=0.3188). Data shown as mean±s.e.m.

One of the main routes of PIP_2_ synthesis is thought to be by PI5K and PI4K-dependent phosphorylation of PI4P and PI(5)P, respectively (Schramp et al., 2015). PI4P was shown to be highly enriched in PC of several cell types (Chávez et al., 2015; Garcia-Gonzalo et al., 2015) and under the tight control of INPP5E which seems not to use PI5P as a substrate (Conduit et al., 2017; Kisseleva et al., 2000; Madhivanan et al., 2015; Schramp et al., 2015). Adenoviral expression of a probe specific for PI4P, P4M-SidM (Hammond et al., 2014) tagged with mCherry showed low abundance in the olfactory cilia of the control WT mice (Fig. 3A, top panel). Conversely, in most OSNs, PI4P was highly enriched in the knob (Fig. 3A). We directly compared levels of PI4P in the knobs of WT and *Inpp5e*^osnKO^ by measuring absolute fluorescence intensity. In the *Inpp5e*^osnKO^ OSNs the mean level of PI4P showed a slight but not significant decrease (Fig. 3D; 179±26 units, WT, *n*=94, 3 mice and 143±17 units, KO, *n*=54, 3 mice; *t*-test, t=0.9777, d.f.=146, *P*=0.3298). Besides PIP_2_, INPP5E also dephosphorylates PIP_3_ at even higher efficiency than PIP_2_, generating PI(3,4)P_2_ (Conduit et al., 2012). PI(3,4)P_2_ was measured using ectopic expression of the Tapp1-PH domain (Fukumoto et al., 2017) and was found to be mostly restricted to the knobs with a low level in cilia. Its distribution pattern was not changed by the loss of INPP5E (Fig. 3B). However, quantitative analysis of PI(3,4)P_2_ revealed significant depletion in the OSN knobs of *Inpp5e*^osnKO^ mice (Fig. 3E, 280±11 units, *n*=830, 3 mice, WT; 174±7, *n*=858, 3 mice, KO; *t*-test, t=8.453, d.f.=1686, *P*<0.0001). Finally, to assay the distribution of PIP_3_, we used a GFP-tagged PH domain of Bruxton tyrosine kinase (Btk–GFP), a well-characterized highly selective PIP_3_ lipid probe (Balla, 2013). Similar to both PI4P and PI(3,4)P_2_, PIP_3_ was highly enriched in the OSN knob, with relatively low quantities in cilia (Fig. 3C, upper panel). Several other probes selective for PIP_3_ based on the PH domains of ARNO, Akt and Grp1 proteins showed an identical distribution to Btk–GFP in OSNs (data not shown). Intriguingly, quantitative analysis of PIP_3_ in OSN knobs of *Inpp5e*^osnKO^ showed a significant increase, by nearly 3-fold (Fig. 3F; 668±64 units, *n*=60, 3 mice, WT; 1495±185, *n*=91, 3 mice, KO; unpaired *t*-test, t=3.536, d.f.=149, *P*=0.0005) with very little if any build-up in cilia (Fig. 3C, KO bottom panel). An inert membrane lipid anchor probe MP-mCherry did not show any preferred partitioning in the membrane in OSN knobs in the WT and the KO (Fig. 3G, MP-mCherry, 340±31 units, *n*=46, 3 mice, WT; 378±23 units, *n*=70, 3 mice, KO; *t*-test, t=1.001, d.f.=114, *P*=0.3188).

### *Inpp5e* deficiency does not affect overall lipid integrity of the ciliary membrane

**Fig. 4.**
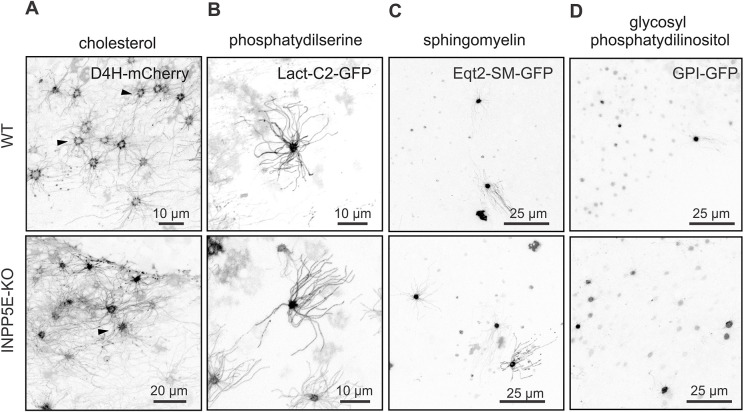
**The distribution of integral membrane lipids was not changed in the OSNs and cilia in *Inpp5e*^osnKO^ KO mice.** (A) D4H–mCherry, a cholesterol-binding probe was enriched in the proximal segment of olfactory cilia equally in the WT and KO OSNs (arrowheads). Cholesterol was also detected, albeit at a lower level, in the full length of the ciliary axoneme. (B) Phosphatidylserine, probed with C2 motif of lactadherin, was uniformly distributed along the cilia and was also enriched in the dendritic knobs of OSNs. (C) A sphingomyelin-specific probe, Eqt2-SMP–GFP, was mostly enriched in the OSN knobs and detected at a low level in cilia. (D) Glycosylated phosphatidylinositol was probed in OSNs with a human folate 1 receptor, GPI–GFP which failed to detect any presence in cilia and it was mostly restricted to the knobs in both the WT and *Inpp5e*^osnKO^ mouse.

We hypothesized that the loss of INPP5E activity resulting in a substantial remodeling of ciliary PIP_2_ may impose an additional effect on overall ciliary lipid composition. We first asked whether cholesterol, which is required for organizing membrane PIP_2_-rich domains, may itself be reciprocally affected by its enrichment. The D4H fragment of bacterial toxin perfringolysin-O recognizing cholesterol in inner membrane leaflet, tagged with mCherry (Maekawa and Fairn, 2015) selectively decorated proximal segments of cilia in WT mice (Fig. 4A, upper panel, arrowheads). Although D4H–mCherry was enriched in the proximal segment, it did label the full length of cilia albeit not as intensely as the MP-mCherry probe. Consistent with the localization of cholesterol, YFP–CAV-1 (a cholesterol binding protein) was also highly restricted to the proximal segment (Fig. S4). Membrane enrichment of PIP_2_ in the *Inpp5e*^osnKO^ however, did not affect overall localization of D4H–mCherry and YFP–CAV-1. This suggests only nominal crosstalk between PIs and cholesterol in olfactory cilia (Fig. 4A, bottom panel; Fig. S4).

A second phospholipid class particularly enriched in the inner leaflet of the plasma membrane and which regulates the trans-bilayer distribution of cholesterol is phosphatidylserine (Maekawa and Fairn, 2015). In accord with the localization of cholesterol probe D4H-mCherry, ectopically expressed phosphatidylserine sensor Lact-C2-GFP, a fragment of lactadherin, was enriched in knobs and in addition evenly distributed along the entire length of cilia (Fig. 4B). Similar to cholesterol, this pattern was not affected in *Inpp5e*^osnKO^ mice (Fig. 4B).

We completed this screen by probing lipids relevant to protein trafficking and targeting, namely, sphingomyelin and glycosylphosphatidylinositol (GPI) (Deng et al., 2016; Paladino et al., 2008). Eqt2-SM–GFP, which contains equinatoxin-2 from the sea anemone *Actinia equina*, is a probe for sphingomyelin, which is associated with Golgi-to-membrane vesicle trafficking (Deng et al., 2016). GPI-anchored proteins (e.g. human folate receptor-1) are directly targeted to the apical membrane in polarized cells, preferentially partitioning into cholesterol-rich raft domains (Paladino et al., 2008). Eqt2-SM–GFP showed partial enrichment of sphingomyelin in cilia of both WT and *Inpp5e*^osnKO^ mice (Fig. 4C) whereas GPI–GFP was highly restricted only to the dendritic knobs in OSNs (Fig. 4D).

### Loss of *Inpp5e* impacts ciliary localization of PIP_2_-binding proteins

**Fig. 5.**
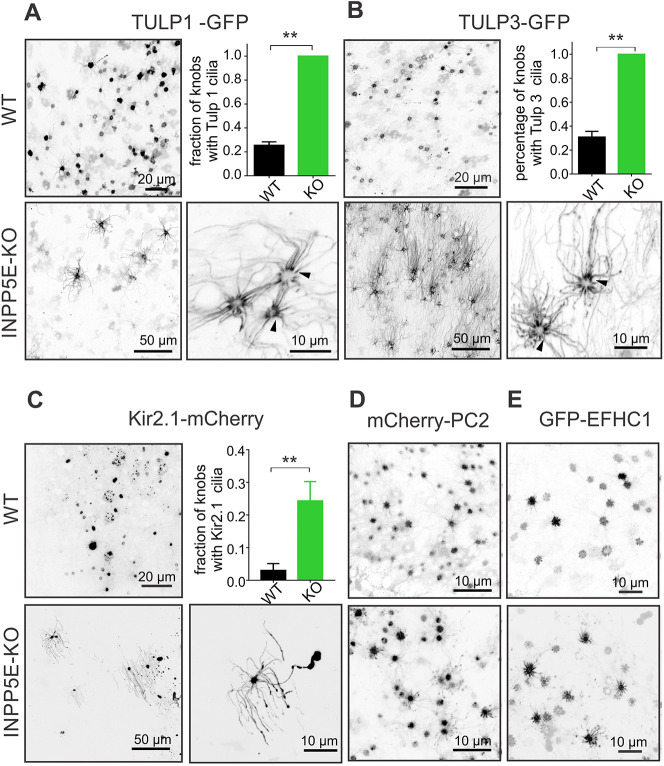
**Soluble and polytopic proteins with affinity to PIP_2_ mislocalize in olfactory cilia of *Inpp5e*^osnKO^.** (A) Tubby-like proteins tagged with GFP (TULP1–GFP and TULP3–GFP) were preferentially restricted to the knobs in the WT (upper left panel). Build-up of PIP_2_ in cilia of the KO resulted in complete redistribution of TULP1 (bottom panels). Note that loss of INPP5E activity led to a depletion of TULP1 within knobs, revealing the proximal segment of cilia decorated with TULP1–GFP (arrowheads, right bottom panel). Quantification of the percentage of the OSN knobs having TULP1-positive cilia per analyzed image showed significant increase in the KO (25.49±0.06%, *n*=4, 3 mice, WT; 100%, *n*=6, 3 mice, KO; Mann–Whitney *t*-test, **P=0.0048). (B) TULP3–GFP, like TULP1, also showed dramatic redistribution between the knob and cilia resulting in a significant increase of percentage of knobs with TULP3-positive cilia (30.87±0.12%, *n*=6, 3 mice, WT; 100%, *n*=4, 3 mice, KO; Mann–Whitney *t*-test, **P=0.0095). (C) The K^+^ inward rectifier ion channel Kir2.1-mCherry, a polytopic protein with two membrane-spanning loops and a known affinity to PIP_2_, also changed its ciliary distribution in *Inpp5e*^osnKO^ OSNs. Kir2.1–mCherry moved into the ciliary membrane in a significantly larger fraction of OSNs in the KO (right upper panel, 3.02±0.02%, *n*=12, 3 mice, WT; 24.34±5.89%, *n*=10, 3 mice, KO; Mann–Whitney *t*-test, **P=0.0023). (D,E) As a negative control, we used a different ion channel, PC2 (PKD2 or TRPP1) tagged with mCherry (mCherry–PC2) and a microtubule-binding protein Efhc1 (GFP-Efhc1), both of which did not change their distribution in the knobs of *Inpp5e*^osnKO^ (upper panels, WT; bottom panels, KO). Data shown as mean±s.e.m.

PIP_2_ and PIP_3_ have been long appreciated as regulators of protein localization and function within structurally defined regions in the plasma membrane (Czech, 2000). Recently, several PIP_2_-binding proteins from the Tubby family, which is implicated in ciliogenesis and ciliary protein trafficking, have been shown to be mislocalized in the PC of cells derived from *Inpp5e* knockout mice (Mukhopadhyay et al., 2010). The members of the Tubby-like protein family TULP1 and TULP3 are anchored to the plasma membrane through their C-terminal PIP_2_-binding motif (Mukhopadhyay and Jackson, 2011; Santagata et al., 2001). Therefore, TULP1 and TULP3 proteins were used as secondary PIP_2_ probes (Hammond and Balla, 2015) and also to test whether translocation of proteins with affinity to PIP_2_ occurs in *Inpp5e*^osnKO^ OSNs. Similar to PLCPH distribution, TULP1–GFP and TULP3–GFP were found mostly in the wild-type OSN knobs (Fig. 5A,B, upper left panel). However, in the *Inpp5e*^osnKO^ OSNs, ectopically expressed TULP1–GFP and TULP3–GFP translocated along the full-length axoneme, mimicking the PLCPH redistribution and demonstrating that PIP_2_ binding is sufficient for their ciliary entry (Fig. 5A,B, bottom panels). Notably, TULP1 and TULP3 were particularly enriched in the ciliary proximal segment of *Inpp5e*^osnKO^ OSNs (Fig. 5A,B, right bottom panels, arrowheads). Overall, the percentage of OSN knobs showing TULP1-positive cilia was dramatically increased in the OSNs of *Inpp5e*^osnKO^ mice (Fig. 5A, upper right panel, 25.49±0.06%, *n*=4, 3 mice, WT; 100%, *n*=6, 3 mice, KO; Mann–Whitney *t*-test, *P*=0.0048). The same redistribution of TULP3 was found in cilia of the *Inpp5e*^osnKO^ mice (Fig. 5B, upper right panel, 30.87±0.12%, *n*=6, 3 mice, WT; 100%, *n*=4, 3 mice, KO; Mann–Whitney *t*-test, *P*=0.0095).

Since TULP1 and TULP3 are peripheral membrane proteins, we asked whether a different protein with a more complex polytopic structure and known to bind PIP_2_ could be translocated into olfactory cilia in *Inpp5e*^osnKO^ OSNs. Potassium inward rectifier channels (Kir1.x-6.x), particularly Kir2.x members are endogenously expressed in the olfactory system (Prüss et al., 2003) and depend on binding PIP_2_ for proper gating (Hansen et al., 2011; Hilgemann et al., 2001; Lee et al., 2016; Logothetis et al., 2015). Indeed, ectopically expressed Kir2.1–mCherry was found to be highly localized to the OSN knob (Fig. 5C, WT left panel) whereas in *Inpp5e*^osnKO^ mice Kir2.1-mCherry moved into the ciliary membrane (WT: 3.02±0.021%, *n*=12, 3 mice; KO: 24.34±5.89%, *n*=10, 3 mice; unpaired *t*-test, t=3.658, d.f.=20, *P*=0.0016). In contrast, other resident proteins expressed in PC [EFHC1 and polycystin-2 (PC2; also known as PKD2)] and not known to bind PIP_2_, failed to redistribute to the full length of cilia in *Inpp5e*^osnKO^ mice (Fig. 5D,E). These data suggest there is specificity to the redistribution of proteins into olfactory cilia following membrane remodeling.

Given the putative role of PIP_2_ and Tubby proteins in the localization of GPCRs in primary cilia (Chávez et al., 2015; Mukhopadhyay et al., 2010; Park et al., 2015), we investigated the endogenous localization of an odorant receptor. We assayed endogenous distribution of the olfactory receptor M71 or M72 (M71/72) using *en bloc* immunocytochemical approach. In WT and *Inpp5e*^osnKO^ mice we found very similar homogenous patterns of M71/72 localization along cilia (Fig. S5A,B). Cilia of 20–30 µm in length were observed both in WT and *Inpp5e*^osnKO^ mice, which is consistent with our measurements using live *en face* imaging (e.g. Fig. 1, MyrPalm–mCherry). This suggests that mechanisms regulating odorant receptor trafficking into OSN cilia differ from GPCRs in PC and raises the question as to the functional consequence of PIP_2_ redistribution following loss of INPP5E in OSN cilia.

### Odor-mediated Ca^2+^ response is modulated by *Inpp5e*

**Fig. 6.**
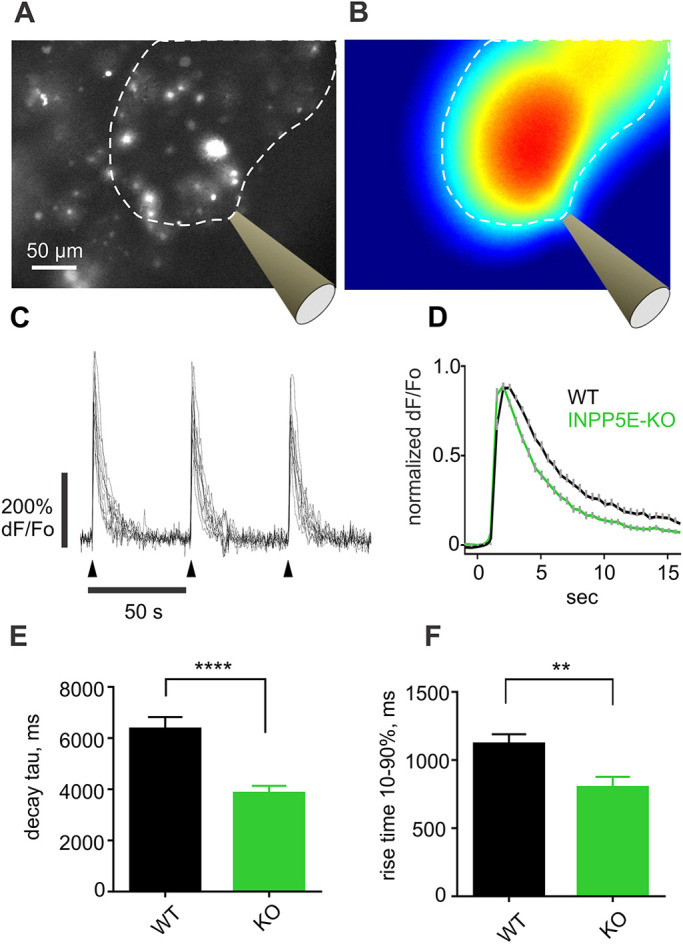
**INPP5E is responsible for shaping the odor-evoked intracellular Ca^2+^ transient in the knob of OSNs.** (A,B) Ectopically expressed GCaMP6F was visualized in the *en face* preparation of mouse OE by wide-field fluorescence microscopy. (A) Bright spots represent numerous OSN knobs. (B) Stimulation micropipette filled with a mixture of 132 different odorants diluted to 1:10,000 in ACSF was positioned as indicated. A single 100-ms pulse at 10 psi pressure generated a plume of fluorescein covering an area over the epithelial surface demarcated by a dotted line. (C) Repetitive application of a single odor pulse (arrowheads) evoked nearly identical responses. GCaMP6F fluorescence corrected for background was calculated as (*F*–*F*o)/*F*o. (D) Individual traces measured in more than 100 OSNs across several areas and 3 mice per each genotype were averaged to create the graph. Traces were normalized to the peak value before averaging. Arrowheads show the time of stimulation. (E,F) The odor-evoked GCaMP6F response had a faster decay in the KO OSNs than the response in the WT control group (WT, 6.49±0.37s, *n*=167, 3 mice; KO, 3.59±0.18s, *n*=110, 3 mice, unpaired *t*-test, t=6.077, d.f.=275, ****P<0.0001). The response in the KO also had a faster rising phase (WT: 1.12±0.07s, *n*=46, 3 mice; KO: 0.80±0.08s, *n*=30, 3 mice, unpaired *t*-test, t=2.936, d.f.=74, **P=0.0044). To calculate termination phase time constant (decay tau) each individual trace was fit to an exponential function. Rise time 10–90% was defined as time to reach from 10% to 90% of the response peak level. Data shown as mean±s.e.m.

Previously, PIs have been established as modulators of ion channels, including olfactory cyclic nucleotide-gated channels (Brady et al., 2006; Hilgemann et al., 2001), and of being involved, specifically, in the control of the odor response of OSNs (Spehr et al., 2002). We hypothesized that unusually high steady-state accumulation of PIP_2_ in cilia as well as elevated PIP_3_ in the OSN knob of *Inpp5e*^osnKO^ mouse may result in altered odor-evoked response. We measured odor-evoked Ca^2+^ transients in the knob of OSNs ectopically expressing the Ca^2+^ indicator GCaMP6f (Fig. 6A–C; Movie 3). A significant decrease of the time constant of termination phase of the GCaMP6f response was observed in *Inpp5e*^osnKO^ compared to the WT OSNs (Fig. 6C–E, decay tau, WT, 6.49±0.37s, *n*=167, 3 mice; KO, 3.59±0.18s, *n*=110, 3 mice, unpaired *t*-test, t=6.077, d.f.=275, *P*<0.0001). Rise time from 10% to 90% of the GCaMP6f odor response amplitude was also significantly shorter in the KO (Fig. 6F, rise time, WT, 1.12±0.07s, *n*=46, 3 mice; KO, 0.80±0.08s, *n*=30, 3 mice, unpaired *t*-test, t=2.936, d.f.=74, *P*=0.0044).

### Odor adaptation is impaired in the *Inpp5e*-deficient mouse

**Fig. 7.**
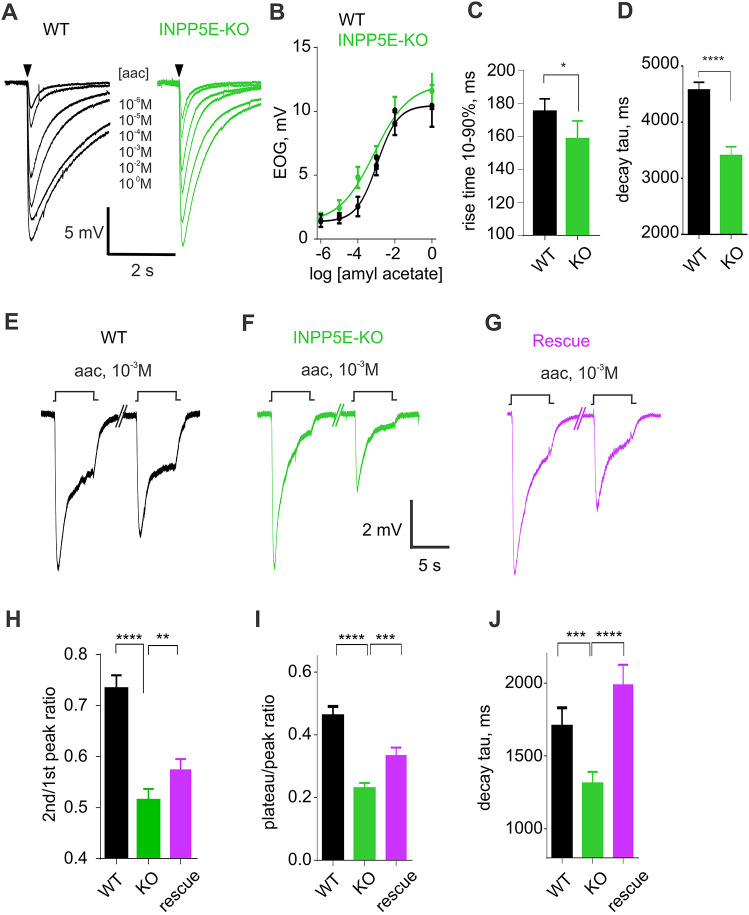
**A faster single-cell odor response translates into a more transient EOG in *Inpp5e*^osnKO^.** (A) Representative EOG traces recorded in response to 100-ms pulse of amyl acetate vapor, driven from the 90-ml head space of bottles containing increasing concentration ranging from 10^−6^ M to a maximum of 1 M (indicated at the individual traces). Odor application is denoted by a black arrowhead. (B) Dose–response relationship showing that there is no significant difference between the WT and KO (WT, *n*=7, 3 mice; KO, *n*=11, 4 mice; two-way ANOVA, *F*(5, 102)=0.1858, *P*=0.9674). (C,D) Rise time of the EOG evoked by a single 100-ms pulse of 10^−2^ M amyl acetate (rise time 10–90%) was decreased in the KO compared to the WT (WT, 174.5±7.7 ms, *n*=37, 5 mice; KO, 157.9±10.9 ms, *n*=40, 7 mice; Mann–Whitney test, *P=0.0221), similar to the time constant (decay tau) of the termination phase (WT, 4.57±0.15s, *n*=81, 5 mice; KO, 3.40±0.16s, *n*=28, 4 mice; unpaired *t*-test, t=4.386, d.f.=107, ****P<0.0001). (E) EOG evoked by a longer 5-s pulse of 10^−3^ M amyl acetate applied at the time indicated by a square step (aac, 10^−3^M) also appeared more transient in the *Inpp5e*^osnKO^ KO (F). Ectopic expression of the full-length WT INPP5E partially rescued the EOG shape (G). (H–J). The ratio between peak amplitude of second and first EOG response, plateau-to-peak ratio and time constant of termination phase (decay tau) were significantly affected by the loss of INPP5E activity and restored by ectopic expression in OSNs of the WT INPP5E. Second/first peak ratio (WT, 0.733±0.026, *n*=18; KO, 0.514±0.022, *n*=9; Rescue, 0.582±0.021, *n*=12; Mann–Whitney *t*-test, WT versus KO, ****P<0.0001; KO versus rescue, *P*=0.0409). Peak/plateau ratio (WT, 0.462±0.028, *n*=11; KO: 0.230±0.017, *n*=13; Rescue: 0.336±0.024, *n*=16; Mann–Whitney *t*-test, WT versus KO, ****P<0.0001; KO versus Rescue, *P*=0.0021). Time constant of termination phase (WT, 1.707±0.124s, *n*=19; KO, 1.311±0.080s, *n*=20; Rescue: 1.991±0.134, *n*=16; Mann–Whitney *t*-test, WT versus KO, *P*=0.0083; KO versus rescue, ****P<0.0001). Data shown in H–J are based on the experiments performed on 9 WT, 6 KO and 6 rescued mice and are presented as mean±s.e.m.

Since Ca^2+^ clearance from cilia and knobs of OSNs is critically involved in shaping the odor response (Stephan et al., 2012), we further analyzed the electrophysiological response to odor in *Inpp5e*^osnKO^ mice. A short 100-ms pulse of amyl acetate vapor of increasing concentration was applied to the freshly dissected olfactory tissue to build a concentration–response curve (Fig. 7A,B). Overall odor sensitivity was not changed in *Inpp5e*^osnKO^ mice [two-way ANOVA, *F*(5, 102)=0.1858, *P*=0.9674] resulting in overlapping dose–response curves (Fig. 7B). However, the kinetics of the response were different in the *Inpp5e*^osnKO^ mice, reminiscent of the changes observed in a single-cell GCaMP6F response. The electroolfactogram (EOG) evoked by 10^−2^ M amyl acetate reached its maximal magnitude faster (Fig. 7C; 10–90% rise time was 174.5±7.7 ms, *n*=37, 5 mice, WT; 157.9±10.9 ms, *n*=40, 7 mice, KO; unpaired Mann–Whitney test, *P*=0.0221). In addition, the response inactivated faster to the baseline (Fig. 7D; termination phase was fit to a single exponential function yielding time constant of 4.57±0.15 s, *n*=81, 5 mice, WT; 3.40±0.16 s, *n*=28, 4 mice, KO; unpaired *t*-test, t=4.386, d.f.=107, *P*<0.0001). Paired-pulse adaptation paradigm did not reveal any difference between the WT and *Inpp5e*^osnKO^ mice using a short 100-ms pulse of amyl acetate (Fig. S3). However, we observed a much stronger effect in *Inpp5e*^osnKO^ mice on adaptation of the EOG response to a repetitive longer 5-s pulse of 10^−3^ M amyl acetate (Fig. 7E,F). Adaptation was measured as the ratio of the peak EOG evoked by the second odor pulse 40 s after the first pulse [Fig. 7E,F, black (WT) and green (KO) traces] and recovered slower in the KO (Fig. 7H,I, second/first peak ratio 0.733±0.026, *n*=18, 9 mice, WT; 0.514±0.022, *n*=9, 6 mice, KO; Mann–Whitney test, *P*<0.0001). The effect of the *Inpp5e* deletion also resulted in a reduced plateau-to-peak ratio (Fig. 7I, ratio of 0.46±0.03s, *n*=11, 9 mice, WT; 0.23±0.02, *n*=13, 6 mice, KO; Mann–Whitney test, *P*<0.0001). Finally, we analyzed decay kinetics by fitting termination phase of the EOG to a single exponential function yielding a time constant of 1707±124 ms, *n*=19, 9 mice, WT and 1311±80 ms, *n*=20, 6 mice, KO (Fig. 7J, Mann–Whitney test, *P*=0.0083). We conclude that deficiency in INPP5E through elevated ciliary PIP_2_ results in a complex sensory exhaustion at the single cell level.

To assay the feasibility of functional rescue, the EOG was measured in adult *Inpp5e*^osnKO^ mice after 10 days of adenoviral ectopic expression of the full-length GFP–INPP5E-FL. A 5-s pulse of 10^−3^ M amyl acetate in the virally treated mice evoked an odor response showing prominent recovery of the adaptation and kinetics (Fig. 7F,G). Statistical analysis confirmed a significant change of the EOG parameters (Fig. 7H–J). The ratio between second/first EOG peak amplitude was increased following rescue treatment [0.514±0.022, *n*=9, 6 mice, KO; 0.582±0.021, *n*=12, 6 mice, Rescue; Mann–Whitney test, *P*=0.0409; one-way ANOVA comparing WT, KO and Rescue groups, *F*(2, 36)=19.85, *P*<0.0001]. Plateau-to-peak ratio was also increased [0.230±0.017, *n*=13, 6 mice, KO; 0.336±0.024, *n*=16, 6 mice, Rescue; Mann–Whitney test, *P*=0.0019; one-way ANOVA comparing WT, KO and Rescue groups, *F*(2, 37)=21.99, *P*<0.0001]. The decay time constant increased in rescued relative to the KO mice [KO, 1.31±0.08 s, *n*=20, 6 mice, KO; 1.99±0.13 s, *n*=16, 6 mice, Rescue; Mann–Whitney test, *P*<0.0001; one-way ANOVA comparing WT, KO and Rescue groups, *F*(2, 52)=9.134, *P*=0.0004]. Together, our findings provide a compelling evidence of the role of PIs as a modulator of the odor response and their involvement in ciliary biology of native multi-ciliated OSNs.

## DISCUSSION

In the current study, we have shown that, in mature OSNs, INPP5E phosphatase activity in cilia creates a gradient of PIP_2_. The loss of INPP5E activity eliminates PIP_2_ restriction to the TZ of cilia and adjacent OSN knob membrane and results in a concomitant change in the abundance of PI(3,4)P_2_ and PIP_3_ in the OSN knob. Importantly, our multiple lines of evidence converge on the conclusion that PIP_2_ redistribution in olfactory cilia plays a modulatory role in their function, but not a role in building nor maintaining cilia, which is different to what is seen with primary cilia or cilia in other systems or cell types. Notwithstanding, the exclusion of PIP_2_ from the full length of cilia in OSNs allows for efficient odor adaptation. It is reasonable to predict that this may translate into an olfactory deficit at the behavioral level. Optimal adaptation kinetics expands the dynamic range of OSNs, thus controlling the acuity of sensory perception (Su et al., 2009). We can envision that faster sensory inactivation in *Inpp5e*^osnKO^ OSNs translates into an impairment of mice to find an odor source in the presence of a background of the same odor, similar to what occurs in the *Cfap69* mutant mouse (Talaga et al., 2017). Therefore, a more detailed study of the odor-driven behavior of *Inpp5e*^osnKO^ mouse, particularly to challenging olfactory tasks, may address these questions in the future.

PIs, such as PIP_2_ and PIP_3_, are implicated in regulation of a vast array of proteins including ion channels and transporters in a tightly regulated spatio-temporal manner (Hilgemann et al., 2018; Hille et al., 2015). A role for PIs, in mammalian olfactory transduction has long been suggested either as second messengers or as constituents of the membrane in which odorant signaling complexes reside (Spehr et al., 2002; Ukhanov et al., 2016). PIs, which under normal conditions are relatively minor components of the membrane, can directly modulate olfactory signaling proteins like the CNG channel or the olfactory Cl^−^ channel TMEM16B (Dibattista et al., 2017; Ta et al., 2017; Zhainazarov et al., 2004). For example, odors may generate a transient change of PIP_2_ and PIP_3_, which has been directly implicated in inhibiting the output of OSNs (Ukhanov et al., 2010, 2016). Therefore, we hypothesized that redistribution of PIP_2_ in OSN cilia of *Inpp5e*^osnKO^ mice would affect the ability to transduce odor signals. Surprisingly, upon disruption of the gradient of PIP_2_ and its steady-state enrichment in *Inpp5e*^osnKO^ OSN cilia, EOG amplitudes were not altered but the response kinetics was accelerated. The more transient EOG response resulted from an acceleration of both the rising phase and termination of the odor response, which may lead to an associated sensory exhaustion. Therefore, the exclusion of PIP_2_ from the full length of cilia would function to slow odor response kinetics. Indeed, a number of proteins outside the principal signaling components are known to modulate olfactory signaling (Buiakova et al., 1996; Kaneko-Goto et al., 2013; Talaga et al., 2017). To our knowledge the slowdown of odor response kinetics has only been measured with one of these protein modulators, namely, cilia- and flagella-associated protein 69 (CFAP69). CFAP69 is an evolutionarily conserved protein localized to OSN cilia and shown to dampen kinetic responses to odors (Talaga et al., 2017). Given the overlapping functional phenotype, it is therefore tempting to speculate that there exists a dynamic reciprocity between CFAP69, or perhaps other orphan house-keeping proteins included in olfactory cilia proteome (Klimmeck et al., 2008; Kuhlmann et al., 2014; Mayer et al., 2009), and ciliary membrane PIs.

Our data show that redistribution of PIP_2_ into the full length of OSN cilia enhanced the rate of Ca^2+^ extrusion from OSNs following odor stimulation. Therefore, an alternative or complementary mechanism for the functional effects resulting from loss of INPP5E in OSN cilia, could be derived from the role of PIP_2_ as a positive regulator of the Na^+^-Ca^2+^ exchanger, as occurs in cardiac cells (He et al., 2000). Ca^2+^ extrusion is important for odor response recovery and short-term adaptation in OSNs (Saidu et al., 2009; Stephan et al., 2012; Zufall and Leinders-Zufall, 2000). However, PIP_2_ build-up in cilia did not cause any adverse effect on overall odor sensitivity, maximal odor evoked EOG or short-term adaptation (Fig. S5C,D). Instead, it impaired a prolonged form of adaptation induced by 5-s odor pulses. Functionally, our finding parallels earlier evidence on the role of CaMKII kinase, which controls a long-form of adaptation (Leinders-Zufall et al., 1999). CaMKII is not known to be directly modulated by PIs; however, it is certainly possible that indirect modulation of CaMKII occurs with redistribution of PIP_2_. Such indirect modulation is possible if perturbation of membrane PIP_2_ disrupted organization of ciliary membrane microdomains, such as rafts, that may be important for spatio-temporal dynamics of Ca^2+^. Lipid rafts are thought to be crucial in organizing ion channels and other signaling molecules, including CaMKII, near the membrane (Hammond, 2016). However, in *Inpp5e*^osnKO^ mice, we did not measure any ciliary alterations in other membrane lipids nor did we detect an alteration in the ciliary localization of odor signaling proteins. Nevertheless, the exclusion of PIP_2_ from the full length of OSN cilia contributes to a unique membrane compartment that is optimized for odor detection.

In addition to a possible direct effect of membrane lipids on ion channels or transporters within the OSN cilium, it is possible that the functional phenotype in OSNs results from perturbation of signaling pathways. Loss of INPP5E not only disrupted cilia localization of PIP_2_ but also elevated PIP_3_ in the plasma membrane of the OSN knob and likely in other cellular compartments of OSNs given that INPP5E is localized to Golgi (Kong et al., 2000). Sustained elevation of PIP_3_ may affect several targets involved in important homeostatic mechanisms, including Akt signaling in close proximity to primary cilia (Hakim et al., 2016). There is evidence for Akt signaling to function in OSNs, where it was shown to be activated under prolonged odor exposure promoting neuronal survival (Kim et al., 2015). In other neurons, disruption of Akt signaling can cause an abnormality in axonal growth, resulting in the ciliopathy Joubert syndrome (Guo et al., 2019). While we did not observe any measurable changes in innervation in the bulb (data not shown), it does not exclude the possibility for perturbation of Akt signaling or other pathways to modulate the electrophysiological properties of OSNs. Therefore, the precise mechanism linking alterations in membrane phosphoinositides in OSN cilia to the cellular odor response should be addressed in future studies.

This is the first report to show the localization of lipid species representing broad classes of membrane lipids in olfactory cilia. One of the things that stood out in our analysis of WT mice was the stochastic variation in the number of neurons and cilia with PIP_2_. While PIP_2_ was largely excluded from the full length of cilia in the majority of neurons, there was a small number of neurons scattered throughout the OE that showed PIP_2_ in a subset of their cilia (Fig. 1A; Fig. S1B). Often these multi-ciliated neurons would have a single cilium in which PIP_2_ was distributed along the membrane of the full length of the axoneme. This was not observed with the other lipid species we analyzed (Figs 3 and 4). The reason for this is unclear but it may reflect the maturation state of the neurons, which are renewed on average every several months (Mackay–Sim and Kittel, 1991). Alternatively, this may represent a subpopulation of neurons expressing unique odorant receptors or a subclass of neurons expressing noncanonical odor signaling components (e.g. GCD, TRPC2 and TAAR receptor neurons) (Munger et al., 2009). Regardless, genetic deletion of INPP5E normalized the distribution of PIP_2_ and caused a redistribution of the lipid to the full length of cilia in all cells.

Our results on the remodeling of PIP_2_ in OSN cilia correlate well with previously published studies on the role of INPP5E in the PC on different cell types of mammalian, fish and insect origin (Chávez et al., 2015; Garcia-Gonzalo et al., 2015; Park et al., 2015; Xu et al., 2017). However, there are several distinct differences for which olfactory cilia may be unique. One outstanding question is the substrate for INPP5E in WT OSN cilia. In primary cilia, PI4P is enriched in the basal state and is decreased with deletion of INPP5E (Chávez et al., 2015; Garcia-Gonzalo et al., 2015). This suggests the INPP5E uses PIP_2_ itself as a substrate in PC. In contrast, we did not measure significant levels of PI4P in the OSN cilia and hence no change was measured in *Inpp5e*^osnKO^ mice. Importantly, PI4P and PIP_2_ comprise two nearly independent pools in the membrane, and previous studies have shown that PI4P may exist in the membrane for a short time as intermediate product channeled from the kinases PI4K to PI5K to make PIP_2_ (Fairn and Grinstein, 2012). Because of this very dynamic process, a steady-state level of PI4P may stay at a nearly undetectable low concentration, which may be the case in the cilia of mammalian OSNs. Instead, our data suggest that in the knob or at the base of cilia, INPP5E uses PIP_3_ as a substrate to make PI(3,4)P_2_. Both lipid species are enriched in the OSN knob, and we found that PIP_3_ levels decreased *Inpp5e*^osnKO^ mice. In the PC of medulloblastoma, INPP5E is mostly involved in converting PIP_3_ into PI(3,4)P_2_ (Eramo and Mitchell, 2016). The potential for dynamic exchange of lipids from the knob, the proximal segment/TZ and full-length cilia in OSNs requires further investigation. Nonetheless, we found that the steady-state ciliary distribution of PIs other than PIP_2_ was not significantly changed in *Inpp5e*^osnKO^ mice. One explanation is that due to redundancy between multiple 5′-phosphatases, resting levels of PI species level are differentially affected by deletion of INPP5E. This is observed in other systems where a combined knockdown of several isoforms of 5′-phosphatases, SHIP1, SYNJ1 and SYNJ2, OCRL and INPP5B, was required to reveal significant elevation of PIP_3_ with only a slight decrease of PI(3,4)P_2_ (Malek et al., 2017). Given that other INPP5E class phosphatases co-exist in OSNs along with INPP5E (Kanageswaran et al., 2015), it would be imperative in future studies to address the growing complexity of the PI pathways in ciliogenesis and ciliary signaling in olfactory system through its development.

There are other aspects of divergence, related to PI pathways, between OSN cilia and a PC. In our study, we did not find a major role for the PIP_2_ gradient in OSN cilia formation or maintenance, which is consistent with work in *C. elegans* reporting that increased ciliary PI(4,5)P_2_ levels are not sufficient to remodel sensory cilia morphology (DiTirro et al., 2019). Other studies, however, have shown that the loss of INPP5E shortened the PC, suggesting a complex INPP5E-dependent regulation of ciliogenesis and maintenance (Chávez et al., 2015; Jacoby et al., 2009; Nozaki et al., 2017; Phua et al., 2017). The formation and functioning of cilia as a cellular organelle is maintained by IFT. In PC, there are well defined interactions between the retrograde IFT-A machinery, adaptor proteins and PIP_2_. For example, the ciliary transport of the receptor GPR161 depends on IFT-122 protein binding to TULP3, which is in turn recruited to the membrane by PIP_2_ (Boubakri et al., 2016; Mukhopadhyay et al., 2010). This mechanism is also responsible for proper ciliary trafficking of mechanosensitive ion channels NompC and PKD2 in *Drosophila* and *C. elegans*, respectively (Bae et al., 2009; Mukhopadhyay et al., 2010; Park et al., 2015). Furthermore, BBSome core proteins, which are directly involved in IFT through interaction with kinesin and dynein motors, are able to bind *in vitro* to several PIs with highest affinity to PI(3,4)P_2_ (Jin et al., 2010). Our previous work has demonstrated that the BBSome functions as a bona fide constituent of IFT in OSN cilia (Uytingco et al., 2019; Williams et al., 2014). Therefore, it was surprising that we did not find any abnormality in the velocity of IFT-A-dependent transport of IFT122 particles or its accumulation inside olfactory cilia of *Inpp5e*^osnKO^ mice (Fig. S2). IFT-B related trafficking of IFT88 appeared to be also unaltered, with a similar particle velocity to that published previously for the wild-type OSNs (Uytingco et al., 2019; Williams et al., 2014). Directly related to this finding, we report no alteration in abundance or ciliary localization of endogenous olfactory receptor M71/72 (Fig. S5). This notion is supported by the lack of any effect of INPP5E deletion on the overall odor sensitivity in the *Inpp5e*^osnKO^ mice. In PC, PIP_2_ is strongly implicated in the trafficking of GPCRs through a INPP5E/TULP3/IFT-A axis (Garcia-Gonzalo et al., 2015; Maurya et al., 2017; Mukhopadhyay et al., 2017). In addition, hedgehog signaling has been suggested to participate in the ciliary localization of mouse odorant receptors (Maurya et al., 2017). However, our results suggest that the trafficking of odorant receptors into OSN cilia is complex and differs from mechanisms of GPCR localization in PC.

In conclusion, our work provides a novel insight into the organization of membrane lipids in cilia of OSNs in normal and disease-related conditions, as well as the functional implications of ciliary membrane lipid perturbation. Ciliopathies associated with altered PIP_2_ distribution are not limited to INPP5E/JBTS but also occur in a similar disease of oculo-cerebro-renal syndrome of Lowe (OCRL). Importantly, the ability to rescue ciliary PIP_2_ distribution and the whole tissue odor response highlight the potential of viral gene therapy treatment for JBTS-related phenotypes in the olfactory system and other impacted tissues.

## MATERIALS AND METHODS

### Mice

All procedures were approved by the University of Florida Institutional Animal Care and Use Committee, protocol 201908162. The *Inpp5e^Δ/flox^* mouse was made in the laboratory of S.S. Mice were housed in a standard animal facility room at the University of Florida. To generate an olfactory tissue specific mutant, we generated homozygous *Inpp5e^flox^*^/flox^ founders, which were crossed with OMP-Cre mice (JAX stock#006668, deposited by Peter Mombaerts). Resulting *Inpp5e*^osnKO^ mice were genotyped using a standard PCR (Jacoby et al., 2009). Mice of both sexes were used in experiments.

### cDNA constructs and adenovirus production

Plasmids containing cDNA fragments were provided as follows: PLCδ1-PH–GFP, Addgene #51407; Btk–GFP, Addgene #51463; mCherry–P4M–SidM, Addgene #51471 (all deposited by Tomas Balla); Tapp1–GFP, a gift from Takeshi Ijuin, Kobe University, Japan; D4H-mCherry, a gift from Gregory Fairn, University of Toronto, Canada; Lact-C2-GFP, Addgene #22852, deposited by Sergio Grinstein; Eqt2-SM–GFP, a gift from Christopher Burd, Yale University, USA; TULP1 and TULP3, a gift from Saikat Mukhopadhyay, University of Texas Southwestern, USA; Kir2.1, Addgene #32669, deposited by Matthew Nolan; PC2 (PKD2), Addgene #83451, deposited by Thomas Weimbs; Efhc1, a gift from Kazuhiro Yamakawa, RIKEN, Japan; IFT122, a gift from Jonathan Eggenschwiler, University of Georgia, USA; GCaMP6f, Addgene #40755, deposited by Douglas Kim. The C-terminal catalytic domain of INPP5E was subcloned from PJ-INPP5E (Addgene #38001, deposited by Robin Irvine). Full-length wild-type human *INPP5E* (NM_019892) was cloned in the lab of S.S. MyrPalm lipid anchored constructs were described previously (Williams et al., 2014). Catalytically dead INPP5E-D477N was made by a site directed mutagenesis of the wild-type gene using a commercial kit (Q5, cat #E0554S, New England Biolabs). All cDNAs were fused with GFP or mCherry, verified by sequencing and subcloned into the pAd/CMV/V5-DEST^TM^ expression vector using Gateway technology (Invitrogen). Adenoviral vectors were propagated in HEK293 cells using the ViraPower protocol (Invitrogen), isolated with the Virapur Adenovirus mini purification Virakit (Virapur, San Diego, CA) and dialyzed in 2.5% glycerol, 25 mM NaCl and 20 mM Tris-HCl, pH 8.0 (Slide-A-Lyzer Dialysis Cassette, 10,000 MWCO) overnight. Alternatively, purified virus was dialyzed and further concentrated using ultrafiltration device Sartorius Vivaspin-6 (100,000 MWCO).

### Immunodetection of INPP5E

Freshly dissected olfactory mucosa was homogenized on ice in a lysis buffer (150 mM NaCl, 50 mM Tris-HCl pH 8.0, and 1% Triton-X-100 complemented with protease inhibitors) for 20 min. The sample was centrifuged at 11,300 ***g*** for 10 min at 4°C. The supernatant was then used for a protein concentration assay, using the Bradford detergent-compatible assay according to the manufacturer's instructions (Bio-Rad, Hercules, CA). Samples were heated for 2 min at 95°C in SDS loading buffer. A total of 30 μg of cell lysis samples were run on a 4–12% Bis-Tris acrylamide gel (Invitrogen). After electrophoretic transfer to nitrocellulose, membranes were incubated with 5% fat-free milk and then with the anti-INPP5E (Proteintech, 17797-1-AP) or anti-actin antibodies (A5060, Sigma) (diluted 1:500 and 1:1000, respectively). Bound primary polyclonal antibody was detected with a 1:5000 dilution of horseradish peroxidase-conjugated goat anti-rabbit-IgG (Zymed). The Renaissance western blot chemiluminescence reagent was used according to the manufacturer's protocol (Perkin Elmer Life Sciences, Wellesley, MA). Images were captured using the EpiChemi3 Darkroom (UVP, Upland, CA). Intensity of specific bands were measured in NIH ImageJ and presented as a ratio between INPP5E and actin signal.

### Whole-mount immunocytochemistry

Mice were killed by inhalation of carbon dioxide followed by cervical dislocation. Freshly dissected turbinates and septum were drop fixed for 3–4 h on ice in freshly prepared 4% paraformaldehyde in a phosphate-buffered saline (PBS), pH 7.4 supplemented with 20% sucrose. Tubes containing the tissue were carefully placed in a refrigerator at 4°C and left for the duration of fixation without any movement or agitation. This step was critical for the preservation of cilia, which are known to be extremely sensitive to mechanical damage. The tissue was thoroughly washed in PBS and blocked with PBS containing 3% fetal bovine serum, 2% bovine serum albumin and 0.3% Triton X-100 for 2 h at room temperature. The tissue was then incubated with primary antibody against mouse M71/72 olfactory receptor (a gift from Dr Gilad Barnea, Brown University, Providence, USA) raised in guinea pig, diluted 1:1000 in the same blocking solution. Finally, the tissue was incubated with secondary anti-guinea pig-IgG conjugated to Alexa Fluor 568 (1:1000) for 2 h and placed in antifading mounting agent Vectashield (Vector Labs) on the glass coverslip. Specimens were analyzed in an inverted Nikon TiE-PFS-A1R confocal microscope. Images were post-processed using Nikon Elements software (version 4.30) and NIH ImageJ (Wayne Rasband, NIH, http://imagej.nih.gov/ij) and assembled in CorelDraw v.18 (Corel).

### *En face* imaging of adenovirally expressed proteins in live mouse OE

To express genes of interest, 10–20 µl of purified viral construct was intranasally administered to mice ranging between 10 and 40 days of age. Typically, viral delivery was repeated in three consecutive days. At 10 days post infection, mice were anesthetized with CO_2_, rapidly decapitated, and entire turbinates and septum were dissected and kept on ice in a Petri dish filled with freshly oxygenated carbogen-modified artificial cerebrospinal fluid (ACSF) that contained (in mM): 120 NaCl, 25 NaHCO_3_, 3 KCl, 1.25 Na_2_HPO_4_, 1 MgSO_4_, 1.8 CaCl_2_, 15 glucose, 305 mOsm (adjusted with sucrose), pH 7.4. For imaging, a small piece of the OE was mounted in the perfusion chamber (RC-23, Warner Instruments) with the apical surface facing down and analyzed on the Nikon TiE-PFS-A1R confocal microscope equipped with a 60× oil-immersion objective, using preset configuration for acquisition of GFP and mCherry fluorescence. Image acquisition settings were set to avoid pixel saturation and maintained equal when comparing WT control and INPP5E-KO tissue.

For total internal reflection fluorescence microscopy (TIRF) *en face* imaging, virally transduced mice were prepared as above. TIRF imaging was performed on a Nikon Eclipse Ti-E/B inverted microscope equipped with a 100× oil immersion CFI APO TIRF 1.49 NA and an EMCCD camera (iXon X3 DU897, Andor Technology).

### Quantification of *en face* confocal *z*-stacks and measuring IFT velocity in TIRF time-series

Confocal *z*-stacks spanning 5 µm from the uppermost cilia to the dendritic knob of the OSN were flattened using sum intensity projection keeping a 16-bit depth throughout the analysis. Fluorescence was corrected for the background and measured within regions of interest.

ImageJ/FIJI was used to generate line-scan kymographs for measuring particle velocities from imported time series. All time series were corrected for the drift due to any tissue movement using the GPU-enabled NanoJ-SRRF plugin (Laine et al., 2019). After trajectories of particle movement were selected for individual identified cilia, the kymographs were extracted using Kymograph plugin. A velocity was calculated using the equation: *tan*(α*π/180)*b/*c*, where *α* is angle, *b* is calibration in μm per pixel and *c* is exposure time per frame.

### Single-cell GCaMP6f Ca^2+^ imaging of the odor-evoked response

Ca^2+^ imaging was performed as described previously (Ukhanov et al., 2016). Mice of 4–6 weeks of age were used for experiments at 10–14 days after administration of adenovirus encoding GCaMP6f. Tissue was prepared and mounted the same way as described above. The chamber was transferred to the stage of upright microscope Zeiss Axioskop2F equipped with a 40×0.75 NA water-immersion objective lens. Experimental solutions were applied directly to the field of view through a 100 µm diameter needle made of fused silica and connected to the 9-channel Teflon manifold. Each perfusion channel was controlled by the electronic valves (VC-6, Warner Instruments). The Ca^2+^ response presented as an increase of GCaMP6f fluorescence emanating from the OSN knob and underlying dendrite. The tissue was illuminated using a standard eGFP filter cube BP490 nm/535 nm (Omega Optical, USA) and the emitted light was collected at 530 nm (BP 530/20 nm, Omega Optical, USA) by a 12-bit cooled CCD camera (ORCA R2, Hamamatsu, Japan). Both the illumination system (Lambda DG-4, Sutter Instruments, USA) and image acquisition were controlled by Imaging Workbench 6 software (INDEC BioSystems). Before processing, fluorescence intensity was corrected for the background. Each OSN knob was assigned a region of interest (ROI), and changes in fluorescence intensity within each ROI were analyzed and expressed as the peak fractional change in fluorescent light intensity (*F*-*F*o)/*F*o, where *F*o is the baseline fluorescence before odorant application. A stock solution of a complex odorant mixture of 42 distinct chemicals (Ukhanov et al., 2013) was made in DMSO as individual 0.5 M stock and then mixed to final 1:10,000 dilution with ACSF. ACSF supplemented with 0.1% DMSO, the odorant carrier, served as the control solution. All 42 odorous chemicals in the stock solution were at the same concentration of 11.9 mM.

Analysis and graphical presentation of Ca^2+^ imaging data was performed with Imaging Workbench 6 (INDEC), Clampfit 9.2 (Molecular Devices), NIH ImageJ, Microsoft Excel and GraphPad Prism 8.

### Electroolfactogram recording

Mice were anesthetized with CO_2_, rapidly decapitated, and the head split along the cranial midline. Septal tissue was removed to expose olfactory turbinates. Vapor-phase odors were delivered by a pressurized nitrogen line connected to a sealed 100 ml glass bottle and directly injected into a continuous stream of humidified carbogen flowing over the tissue. Odorants were prepared by diluting pure stock into deionized water and final working concentration calculated as a molar value (v/v). Responses to odors were recorded with a standard glass micropipette tip-filled with agarose and backfilled with PBS using a Multiclamp 700A amplifier controlled by Multiclamp 700A and Clampex 9.2 software (Molecular Devices). EOG was measured as the maximal peak amplitude from the pre-pulse baseline using Clampfit 9.2 software (Molecular Devices).

### Statistical analysis

All statistical tests were done in Prism 8 (GraphPad) following test for normality and by using non-parametric Mann–Whitney test, unpaired *t*-test or one-way ANOVA, and *P*<0.05 was considered to be statistically significant. All the group statistics are presented as mean±s.e.m.

## Supplementary Material

Supplementary information

Reviewer comments
