# Supplementary material for: INPP5E controls ciliary localization of phospholipids and the odor response in olfactory sensory neurons
Source: J Cell Sci. 2021 May 7;135(5):jcs258364. doi: 10.1242/jcs.258364 (PMC8126451; doi:10.1242/jcs.258364)
Supplement: Supplementary information [file joces-135-258364-s1.pdf]

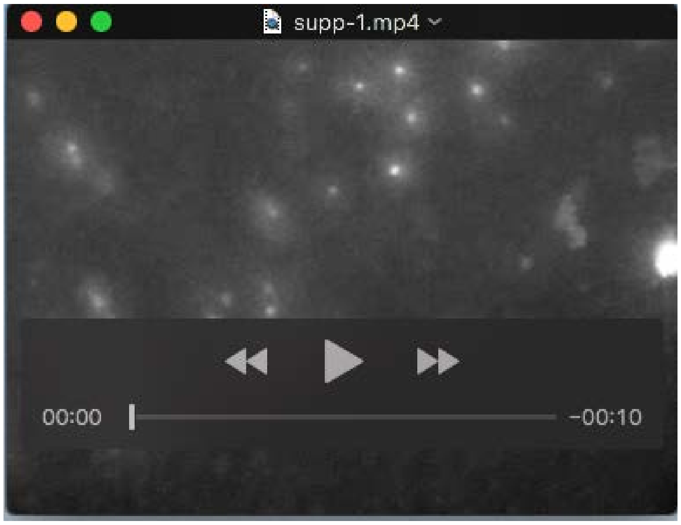

Movie 1. Intraflagellar transport of IFT122 particles in olfactory cilia of INPP5E^{osnKO} mouse. Live *en* face TIRF microscopy used to measure ectopically expressed IFT122-GFP particle movement along cilia in multiple OSNs of *Inpp5e*^{osnKO} mouse. Real time rate is 10 frames per second.

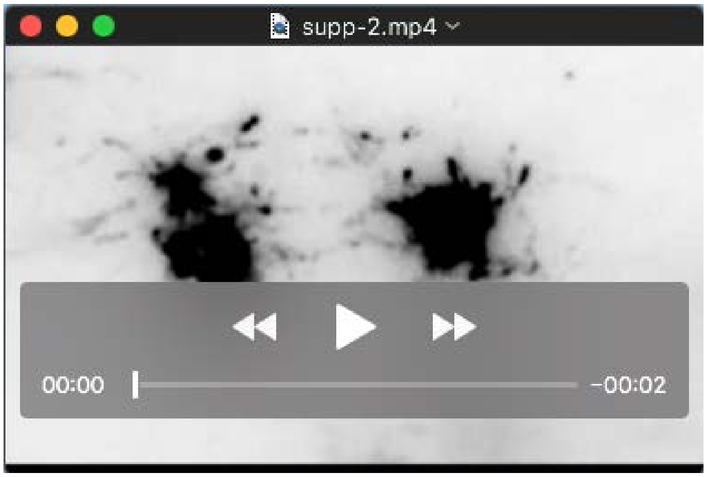

Movie 2. Intraflagellar transport of IFT88 particles in olfactory cilia of INPP5E^{osnKO} mouse. Live *en* face TIRF microscopy used to measure ectopically expressed IFT88-GFP particle movement along cilia in multiple OSNs of *Inpp5e*^{osnKO} mouse. Real time rate is 10 frames per second.

Journal of Cell Science • Supplementary information

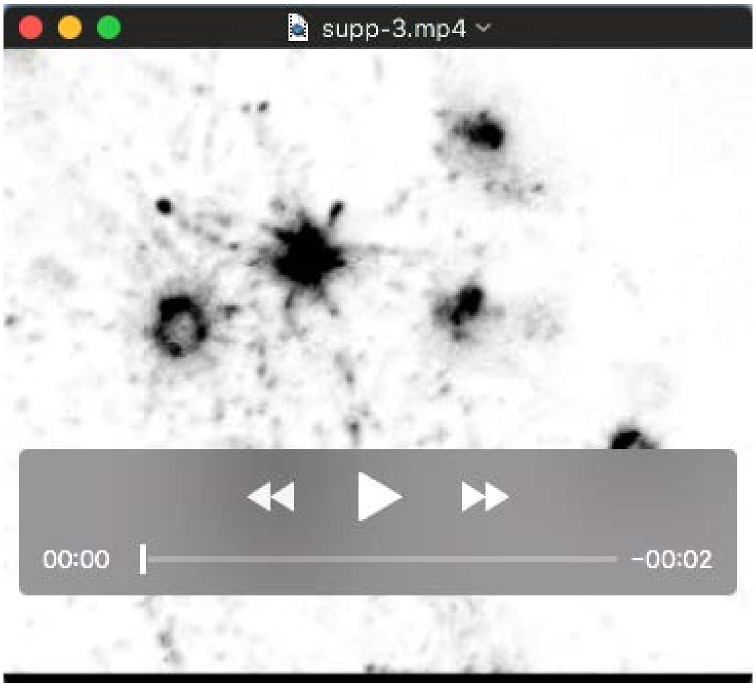

Movie 3. Odor application induces transient elevation of GCaMP6f fluorescence in knobs of mouse OSNs. Ectopically expressed GCaMP6f was used to report stimulus-evoked activity in mouse OSNs. Live *en face* imaging of acutely dissected olfactory epithelium reported change of GCaMP6f fluorescence in the dendritic knobs of *Inpp5e*^{osnKO} OSNs. A 100-ms pressure-pulse was applied from the micropipette filled with ACSF and added odor mix. Total of 5 pulses was applied with 60-s intervals in between. Real time rate is 25 frames per second.

Journal of Cell Science • Supplementary information

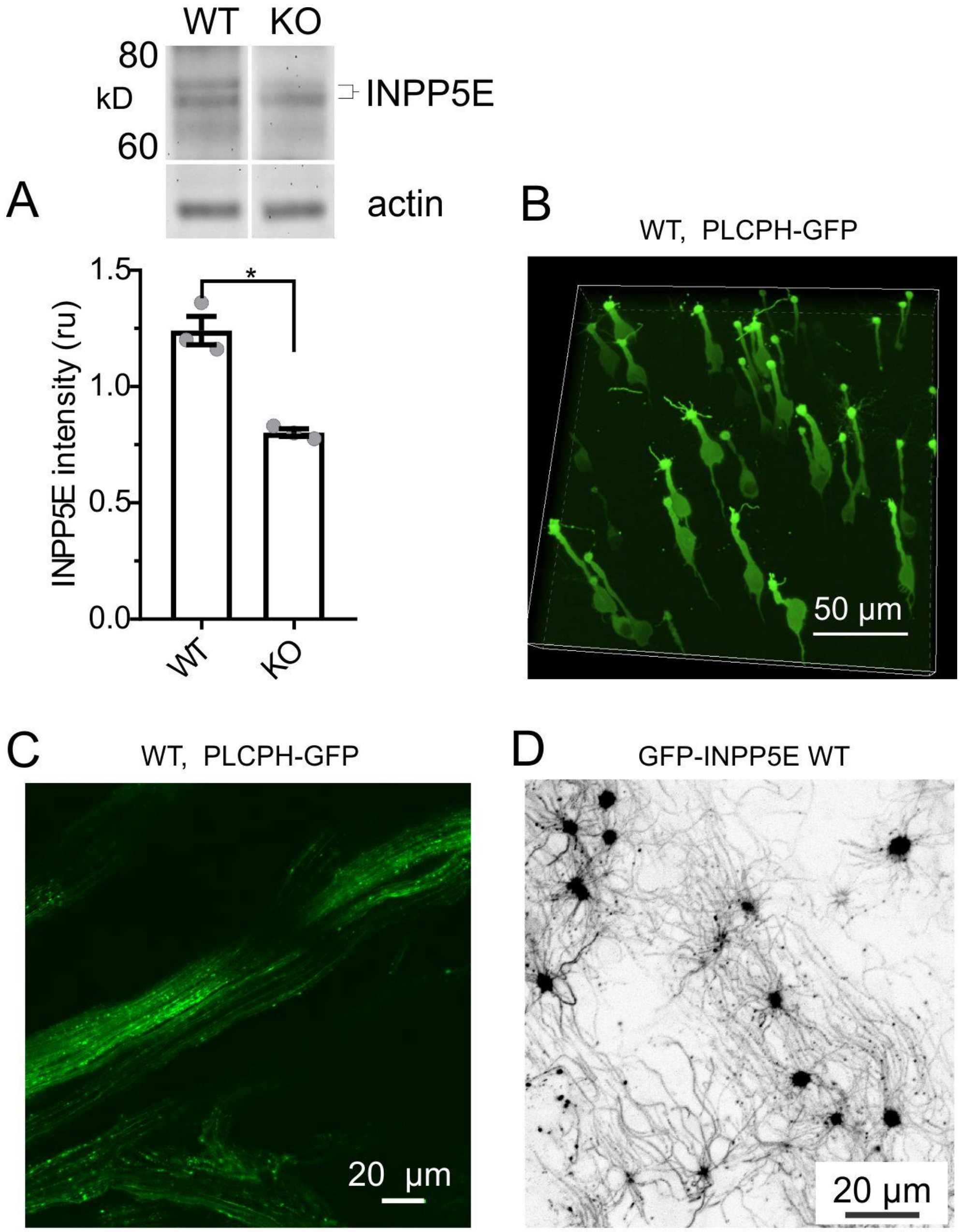

Figure S1

Journal of Cell Science • Supplementary information

Figure S1. Endogenous expression of INPP5E is significantly decreased in the olfactory mucosa of the conditional INPP5E^{osnKO} mice. (A) Double band detected around 70 kD corresponds to the full-length and truncated splice variant of INPP5E (bracket). Intensity of the upper band was calculated as a ratio to the intensity of actin band (shown in relative units, ru). The blot was first probed with anti-INPP5E and then re-probed with anti-actin antibody. Tissue homogenate was prepared from three WT and KO mice (Mann-Whitney unpaired t-test, *p=0.0144, n=3, mean ± SE). (B,C) Distribution of ectopically expressed PI(4,5)P_2 probe, PLCPH-GFP in wild-type mouse OSNs. (B) 3-D volume rendering of the z-stack taken from apical surface of the OE down to the initial segment of axons. (C) PLCPH-GFP decorates plasma membrane of OSNs along the full length of olfactory nerve. An image was taken at the position where the nerve enters cribriform plate and olfactory bulb. (D) Ectopically expressed full-length wild-type INPP5E (GFP-INPP5E WT) is localized to the full length of cilia in WT OSNs.

Journal of Cell Science • Supplementary information

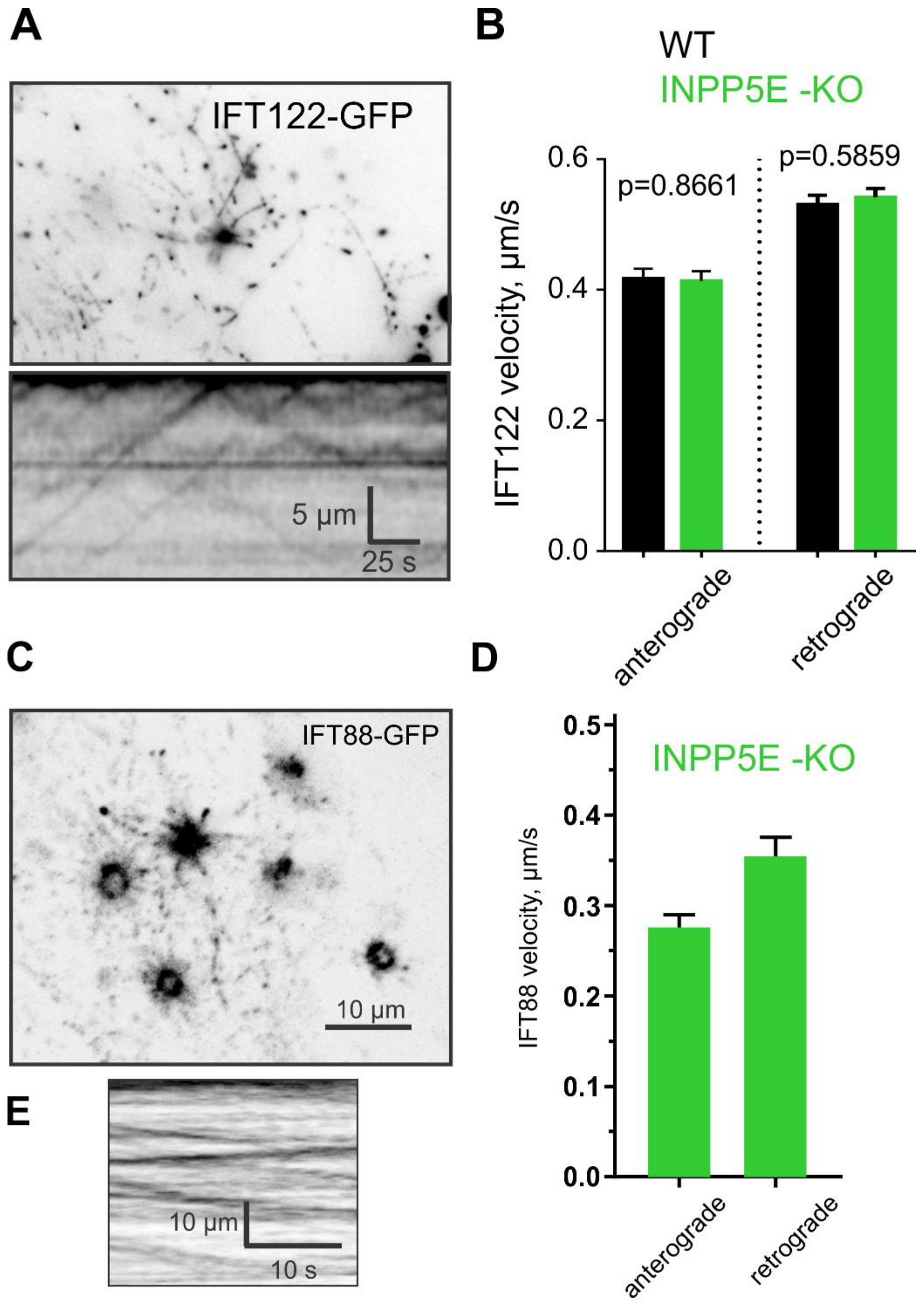

Figure S2

Journal of Cell Science • Supplementary information

Figure S2. Velocity of intraflagellar transport (IFT) was not affected by the loss of INPP5E. (A) IFT particles incorporating ectopically expressed IFT122 protein, tagged with GFP (upper panel) was visualized by TIRF microscopy. Kymograph plot was generated by tracks of moving individual IFT particles (bottom panel). (B) Both the anterograde and retrograde transport of IFT122-GFP particles did not change due to the loss of INPP5E (anterograde IFT: 0.417±0.016 µm/s, n=336, WT, 2 mice; 0.414±0.015 µm/s, n=398, KO, 3 mice; unpaired t-test, t=0.1687 df=732, p=0.8661; retrograde IFT: 0.531±0.014 µm/s, n=360, WT, 2 mice; 0.542±0.014 µm/s, n=469, KO, 3 mice; unpaired t-test, t=0.545 df=827, p=0.5859). (C) A snapshot image of *Inpp5e*^{osnKO} OSNs taken during acquisition of a time-series shows multiple single IFT88-GFP particles distributed along the full length of cilia. (E) Kymograms were generated to measure IFT88 particle velocity in anterograde and retrograde directions in *Inpp5e*^{osnKO}, 0.28±0.15 µm/s (n=101) and 0.36±0.02 (n=83), 2 mice, respectively.

Journal of Cell Science • Supplementary information

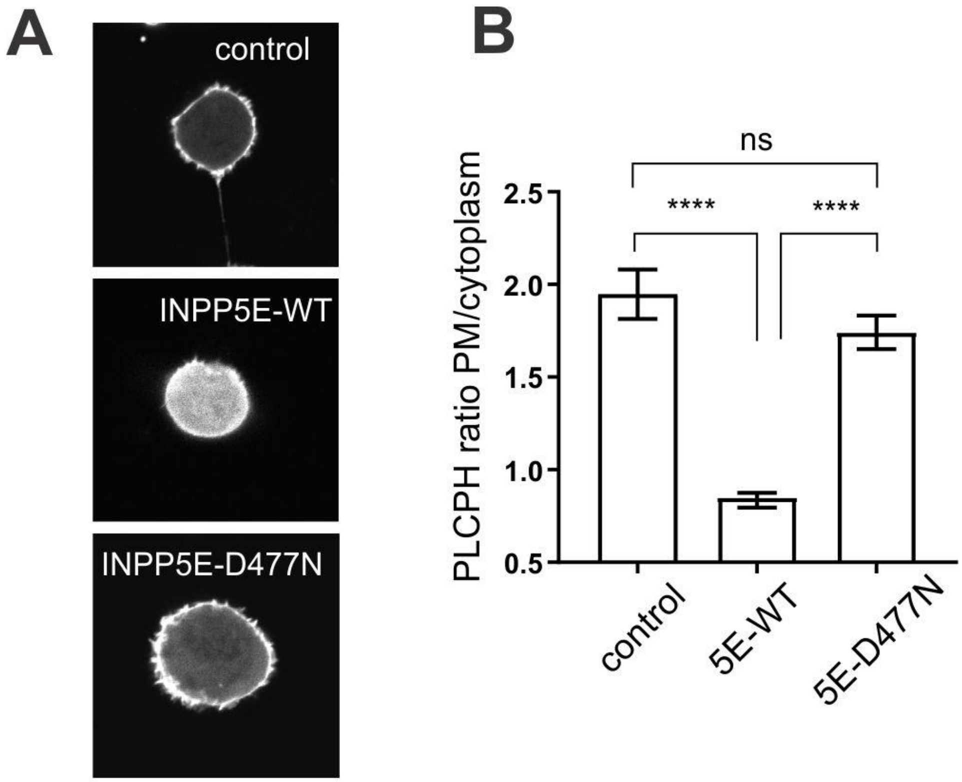

Figure S3

Figure S3. Catalytically inactive point-mutant INPP5E-D477N fails hydrolyzing PIP_2 in the plasma membrane of HEK293 cells. (A) HEK293 cells were transfected with pAd-PLCPH-mCherry (control), and with added pAd-GFP-INPP5E-WT (INPP5E-WT) and pAd-GFP-INPP5E-D477N (INPP5E-D477N) and allowed for expression for 30 h. (B) Fluorescence of PLCPH-mCherry was measured at the plasma membrane and in the cytoplasm. Ratio of the membrane-delimited and cytoplasm intensity was calculated and plotted in three experimental conditions. Co-expression of GFP-INPP5E-WT (5E-WT) induced translocation of PLCPH probe to cytoplasm due to hydrolysis of PIP_2 whereby D477N point-mutant did not. Three groups were compared using a one-way ANOVA and found to be significantly different (F=33.18, (DFn, DFd) = 11.14 (2, 126), ****p<0.0001). Control and D477N groups were not significantly different. Data collected in three independent experiments. Total number of cells used in the experiment, control (n=40), 5E-WT (n=38), and 5E-D477N (n=51).

Journal of Cell Science • Supplementary information

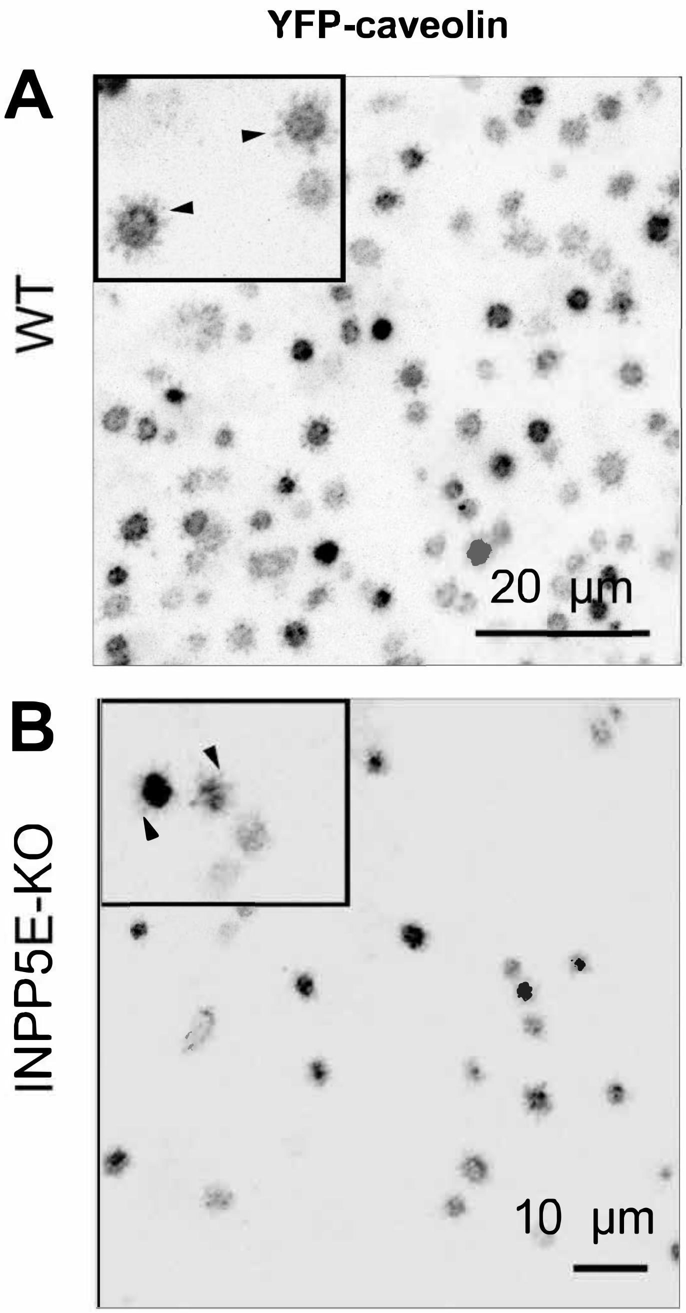

Figure S4

Figure S4. Localization of caveolin-1 in wild-type and *Inpp5e*^{osnKO} OSNs. (A,B) YFP-Caveolin1 was ectopically expressed in mouse OE and exclusively localized to dendritic knobs and decorated also proximal segment (inset, arrowheads) of olfactory cilia.

Journal of Cell Science • Supplementary information

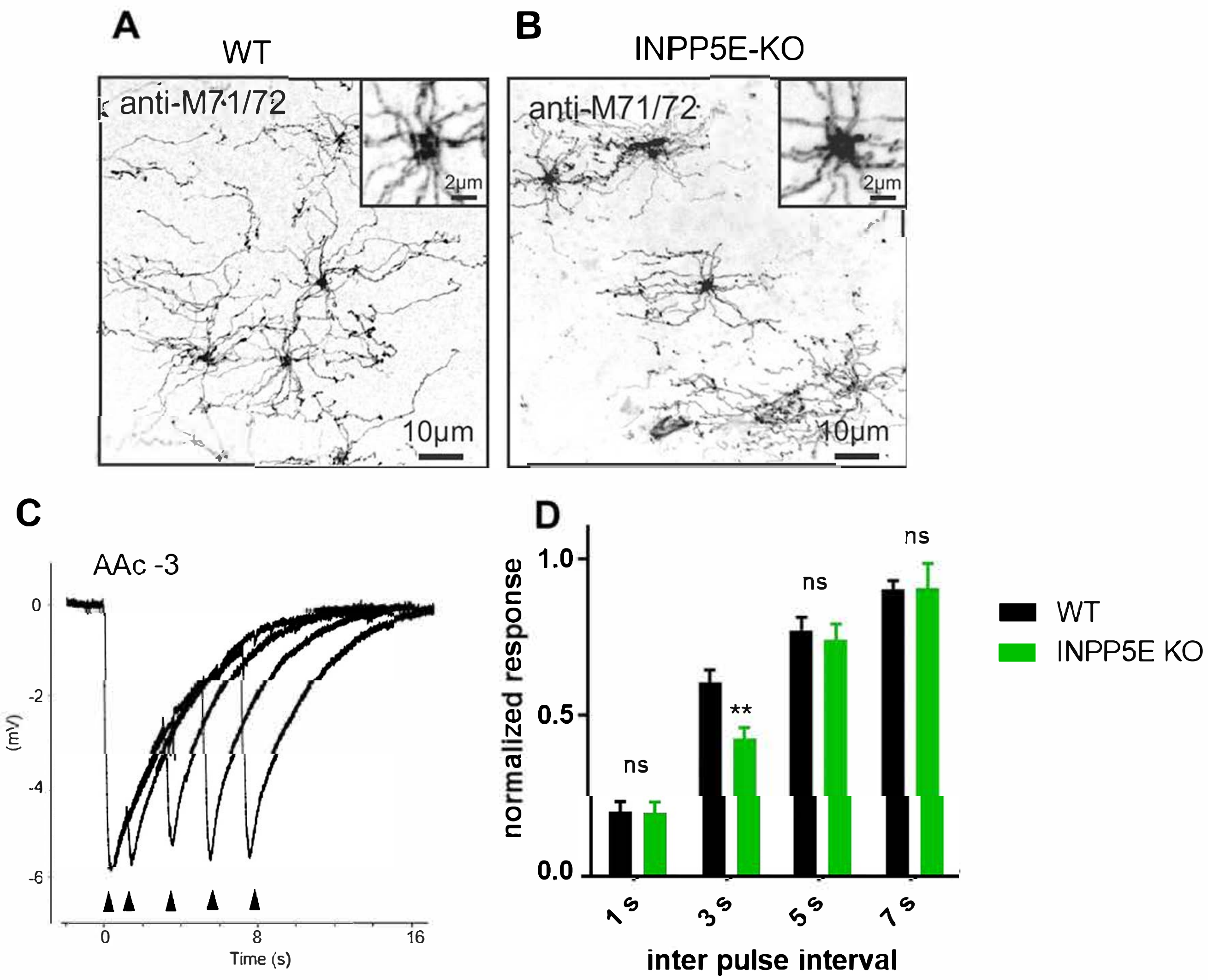

Figure S5

Figure S5. Ciliary expression of endogenous mouse olfactory receptor M71/72 and a short-term adaptation were not affected by the loss of INPP5E. (A,B) *En bloc* immunostaining of the OE with antibody against mouse M71/72 OR. Individual cilia often contained numerous particles due to the fixation artifacts. Also visible are fragments of cilia broken during fixation. Otherwise intensity of labeling and overall distribution of M71/72, also shown in enlarged insets, was unchanged in the WT and *Inpp5e^{osnKO}*. (C) A brief 100-ms test pulse of amylacetate vapor (AAc -3, 10^{-3} M) was applied at time zero followed by a 2nd identical pulse at an indicated time interval. (D) Mean EOG amplitude evoked by the 2nd pulse was plotted at different intervals, showing no difference between WT group, n=12, 5 mice and the KO group, n=12, 4 mice. Slower recovery, however, was detected at 3-s interval. Unpaired Mann-Whitney test, dF=22, **p=0.0027
