# Supplementary material for: INPP5E controls ciliary localization of phospholipids and the odor response in olfactory sensory neurons
Source: J Cell Sci. 2021 May 7;135(5):jcs258364. doi: 10.1242/jcs.258364 (PMC8126451; doi:10.1242/jcs.258364)
Supplement: Reviewer comments [file joces-135-258364_review_history.pdf]

INPP5E controls ciliary localization of phospholipids and odor response in olfactory sensory neurons

Kirill Ukhanov, Cedric Uytingco, Warren Green, Lian Zhang, Stephane Schurmans and Jeffrey R. Martens
DOI: 10.1242/jcs.258364

Editor: James Olzmann

Review timeline
Original submission: 31 December 2020
Editorial decision: 25 January 2021
First revision received: 9 March 2021
Accepted: 15 March 2021

Original submission

First decision letter

MS ID#: JOCES/2020/258364

MS TITLE: INPP5E controls ciliary localization of phospholipids and odor response in olfactory sensory neurons

AUTHORS: Kirill Ukhanov, Cedric Uytingco, Warren Green, Lian Zhang, Stephane Schurmans, and Jeffrey R Martens
ARTICLE TYPE: Research Article

We have now reached a decision on the above manuscript.

To see the reviewers' reports and a copy of this decision letter, please go to: https://submit-jcs.biologists.org and click on the 'Manuscripts with Decisions' queue in the Author Area. (Corresponding author only has access to reviews.)

As you will see, both reviewers found that the data are of high quality and the discoveries provide insights into the role of lipids in the cell biology of olfactory cilia. However, they raise several concerns that prevent me from accepting the paper at this stage. They suggest, however, that a revised version might prove acceptable, if you can address their concerns. The clarifications and experiments requested by the reviewers seem reasonable and important, and I believe that they will help to improve the manuscript and the conclusions. If you think that you can deal satisfactorily with the criticisms on revision, I would be pleased to see a revised manuscript. We would then return it to the reviewers.

We are aware that you may be experiencing disruption to the normal running of your lab that makes experimental revisions challenging.If it would be helpful, we encourage you to contact us to discuss your revision in greater detail. Please send us a point-by-point response indicating where you are able to address concerns raised (either experimentally or by changes to the text) and where you will not be able to do so within the normal timeframe of a revision. We will then provide further guidance. Please also note that we are happy to extend revision timeframes as necessary.

© 2021. Published by The Company of Biologists under the terms of the Creative Commons Attribution License (https://creativecommons.org/licenses/by/4.0/).

Please ensure that you clearly highlight all changes made in the revised manuscript. Please avoid using 'Tracked changes' in Word files as these are lost in PDF conversion.

I should be grateful if you would also provide a point-by-point response detailing how you have dealt with the points raised by the reviewers in the 'Response to Reviewers' box. Please attend to all of the reviewers' comments. If you do not agree with any of their criticisms or suggestions please explain clearly why this is so.

Reviewer 1

Advance summary and potential significance to field

In their manuscript, Ukhanov et al investigate the role of polyphosphoinositides (PIs) in the cell biology of olfactory cilia. They used a number of highly specific molecular probes to determine the PI composition of the ciliary membrane and show that conditional inactivation of Inpp5e in olfactory sensory neurons (OSNs) leads to a redistribution of PI(4,5)P2 along the ciliary membrane and to altered expression of PI(3,4)P2 and PI(3,4,5)P3 in the ciliary knob. These effects could be reversed by ectopic expression of wild-type Inpp5e protein but not of a mutant Inpp5e protein lacking phosphatase activity. Finally, these changes in PI composition correlated with altered odor-evoked electrical responses and odor-induced elevation of cytoplasmatic Ca2+.
Overall, the authors use state of the art techniques to address an important question in ciliary biology. Their findings are supported by beautiful figures and provide novel insights into the role of lipids in the cell biology of olfactory cilia. Therefore, I recommend publication in the Journal of Cell Science, however, the authors need to address some points before acceptance.

Comments for the author

Major points:

1) The authors provide no information on the expression of the Inpp5e protein in OSNs and in particular in their cilia. Moreover, the result section of the manuscript lacks a description of the design used to conditionally inactivate Inpp5e in OSNs and which driver line was used. In particular, no experiment was done to demonstrate that Inpp5e was indeed deleted from the OSNs. The authors should perform immunofluorescence analysis of Inpp5e in control and mutant OSNs to demonstrate where the protein is expressed and whether its expression is lost in the mutants.

2) The authors convincingly show that the Inpp5e mutation dramatically alters the PI composition of the olfactory cilium. This alteration correlates with a change in the electrophysiological properties of the OSNs, however, it remains unclear whether there is a causative relationship between the two findings. Inpp5e has been demonstrated extensively to control multiple signalling pathways including Akt signalling which could affect ion channels or transporters within the OSN cilium. The authors should at least discuss the possibility of alternative pathways which may affect the electrophysiological properties of the OSNs.

3) In Figure 2 and Supplementary Figure S3, the authors use ectopic expression of full-length wild-type Inpp5e or D477N mutant Inpp5e protein to test for a reversal of PI mislocalization in Inpp5e mutant OSNs. The authors should combine both data sets into a single figure since the mutant Inpp5e protein is an important negative control to rule out unspecific effects caused by the ectopic expression.

4) Statistics: For most of their experiments, the authors provide an n number that seems to reflect the number of cells investigated, however, they do not indicate how often experiments were repeated. To avoid pseudo-replication the authors should indicate the number of biological replicates throughout the manuscript and use the average of each replicate for statistical analysis rather than measurements of individual cells.

Typo:

p15: it should read Gpr161

© 2021. Published by The Company of Biologists under the terms of the Creative Commons Attribution License (https://creativecommons.org/licenses/by/4.0/).

Reviewer 2

Advance summary and potential significance to field

Recent studies have established a critical role for phosphoinositides in the regulation of the trafficking of proteins to primary cilia. Moreover dysregulation of ciliary phosphoinositides disrupts ciliary function and is associated with a human ciliopathy. Thus, there is a great deal of interest in understanding the role of phosphoinositides in the function of cilia across diverse cell types. In this study, Ukhanov et al. disrupt INPP5E which is a phosphoinositide phosphatase that is disrupted in patients with Joubert syndrome, specifically in mouse olfactory sensory neurons (OSNs).
They discover that loss of INPP5E leads to redistribution of PIP2 mislocalization of PIP2 binding proteins, and impaired odor adaptation.
Moreover, these defects can be rescued by reintroduction of widtype INPP5E but not catalytically inactive INPP5E. Overall, the manuscript is very well written, the data are high quality, and the results reveal interesting consequences of loss of INPP5E in OSNs and how these consequences vary between other primary cilia.

Comments for the author

One thing that is noticeably absent from the manuscript are data showing whether the KO mice have a detectable olfactory deficit. In lieu of such data the authors could simply speculate about this in the discussion.

Additional points:
1) Page 6, first paragraph, second to last sentence, it appears the results for the mutant and wildtype INPP5E constructs are reversed. Also, they should refer to Fig. S3F, not Fig. 3C-G.
2) Figure 1, it is confusing why the overlapping colors are white in panel A but there is clear green signal in the cilia in panel B. Don’t PLCPH and MP-mCherry overlap in KO cilia?
3) Figure legend 2B, ciliary PIP2 is mCherry not GFP.
4) Figure legend 2D, specify that the last set of values (i.e. 4.2 +/- 0.3 (n=122)) corresponds to the rescue.
5) Figure legend 3, correct the grammar in the heading.

First revision

Author response to reviewers' comments

Authors’ Response (in blue) to Critique of Manuscript # JOCES/2020/258364:

Reviewer 1

Advance Summary and Potential Significance to Field:

In their manuscript, Ukhanov et al investigate the role of polyphosphoinositides (PIs) in the cell biology of olfactory cilia. They used a number of highly specific molecular probes to determine the PI composition of the ciliary membrane and show that conditional inactivation of Inpp5e in olfactory sensory neurons (OSNs) leads to a redistribution of PI(4,5)P2 along the ciliary membrane and to altered expression of PI(3,4)P2 and PI(3,4,5)P3 in the ciliary knob. These effects could be reversed by ectopic expression of wild-type Inpp5e protein but not of a mutant Inpp5e protein lacking phosphatase activity. Finally, these changes in PI composition correlated with altered odor-evoked electrical responses and odor-induced elevation of cytoplasmatic Ca2+. Overall, the authors use state of the art techniques to address an important question in ciliary biology. Their findings are supported by beautiful figures and provide novel insights into the role of lipids in the cell biology of olfactory cilia. Therefore, I recommend publication in the Journal of Cell Science, however, the authors need to address some points before acceptance.

© 2021. Published by The Company of Biologists under the terms of the Creative Commons Attribution License (https://creativecommons.org/licenses/by/4.0/).

Reviewer 1 Comments for the Author:

Major points:

1) The authors provide no information on the expression of the Inpp5e protein in OSNs and in particular in their cilia. Moreover, the result section of the manuscript lacks a description of the design used to conditionally inactivate Inpp5e in OSNs and which driver line was used. In particular, no experiment was done to demonstrate that Inpp5e was indeed deleted from the OSNs. The authors should perform immunofluorescence analysis of Inpp5e in control and mutant OSNs to demonstrate where the protein is expressed and whether its expression is lost in the mutants.

1) We thank the reviewer for identifying that we omitted a description of the design to conditionally inactivate INPP5E. We apologize for this oversight and now provide the details of the olfactory-specific knockout of INPP5E in the Results section.

We appreciate the reviewer's comment to show INPP5E expression in OSNs from wildtype and mutant animals. We now provide Western Blot data (representative images and densitometry) of OE extracts in supplemental Figure S1A, that shows protein expression of a doublet at ~72kD corresponding to INPP5E wildtype and splice variant (Jacoby et al., 2009) that is decreased in the mutant animals. We recognize that this is supportive, but perhaps not conclusive, evidence for a deletion of INPP5E in OSN cilia given that there are multiple cell types that comprise the OE. We did attempt immunodetection of INPP5E on cryosections of the mouse OE but our data indicate that the antibody labeling was non-specific in the olfactory tissue. Nonetheless, we now acknowledge these points in the text of the manuscript (line 131- 139). It is also worth noting that it is well appreciated by the olfactory field that number of important proteins implicated in sensory function and maintenance of OSNs detected at the mRNA level or in proteins isolated from the OE, fail immunodetection in cilia per se with existing antibodies. We do however, now cite data from the literature that shows INPP5E transcripts are expressed in OSNs and cilia proteomic data indicating the presence of INPP5E protein in OSN cilia (lines 135-139). We also highlight that the phenotype (redistribution of cilia PIP2) for loss of INPP5E is consistent with other cells/tissues. Importantly, this phenotype is rescued in OSNs by restoration of wildtype INPP5E but not the catalytically inactive mutant (Fig. 2).

2) The authors convincingly show that the Inpp5e mutation dramatically alters the PI composition of the olfactory cilium. This alteration correlates with a change in the electrophysiological properties of the OSNs, however, it remains unclear whether there is a causative relationship between the two findings. Inpp5e has been demonstrated extensively to control multiple signalling pathways including Akt signalling which could affect ion channels or transporters within the OSN cilium. The authors should at least discuss the possibility of alternative pathways which may affect the electrophysiological properties of the OSNs.

2. We agree with the reviewer and think this is a good suggestion. We have added text to the Discussion of the manuscript (lines 400-414) to discuss the possibility of alternative pathways, such as Akt signaling, that could affect ion balance in OSNs.

3) In Figure 2 and Supplementary Figure S3, the authors use ectopic expression of full-length wild-type INPP5E or D477N mutant Inpp5e protein to test for a reversal of PI mislocalization in Inpp5e mutant OSNs. The authors should combine both data sets into a single figure since the mutant Inpp5e protein is an important negative control to rule out unspecific effects caused by the ectopic expression.

3. We agree with the critique and combined the data in Figure 2.

4) Statistics: For most of their experiments, the authors provide an n number that seems to reflect the number of cells investigated, however, they do not indicate how often experiments were repeated. To avoid pseudo-replication the authors should indicate the number of biological replicates throughout the manuscript and use the average of each replicate for statistical analysis rather than measurements of individual cells.

© 2021. Published by The Company of Biologists under the terms of the Creative Commons Attribution License (https://creativecommons.org/licenses/by/4.0/).

4. We apologize for this oversight. The Number of animals as a measure of independent replicates used to collect respective datasets were added.

Typo:

p15: it should read Gpr161
Thank you for discovering this type on p15: It was corrected

Reviewer 2

Advance Summary and Potential Significance to Field:

Recent studies have established a critical role for phosphoinositides in the regulation of the trafficking of proteins to primary cilia. Moreover, dysregulation of ciliary phosphoinositides disrupts ciliary function and is associated with a human ciliopathy. Thus, there is a great deal of interest in understanding the role of phosphoinositides in the function of cilia across diverse cell types. In this study, Ukhanov et al. disrupt INPP5E, which is a phosphoinositide phosphatase that is disrupted in patients with Joubert syndrome, specifically in mouse olfactory sensory neurons (OSNs). They discover that loss of INPP5E leads to redistribution of PIP2, mislocalization of PIP2 binding proteins, and impaired odor adaptation. Moreover, these defects can be rescued by reintroduction of widtype INPP5E, but not catalytically inactive INPP5E. Overall, the manuscript is very well written, the data are high quality, and the results reveal interesting consequences of loss of INPP5E in OSNs and how these consequences vary between other primary cilia.

Reviewer 2 Comments for the Author:
One thing that is noticeably absent from the manuscript are data showing whether the KO mice have a detectable olfactory deficit. In lieu of such data, the authors could simply speculate about this in the discussion.

We agree with the reviewer's comment. This issue has been now discussed in the manuscript in the Discussion (lines 345-352).

Additional points:
1) Page 6, first paragraph, second to last sentence, it appears the results for the mutant and wildtype INPP5E constructs are reversed. Also, they should refer to Fig. S3F, not Fig. 3C-G.

Figures 2 and S3 were revised as suggested by both reviewers.

2) Figure 1, it is confusing why the overlapping colors are white in panel A but there is clear green signal in the cilia in panel B. Don't PLCPH and MP-mCherry overlap in KO cilia?

We purposely created in Figure 1 (middle panel) a color shifted overlap image to point at the PIP2 domain extended to the full cilia length in this neuron. The figure caption was revised accordingly.

3) Figure legend 2B, ciliary PIP2 is mCherry not GFP.

Corrected

4) Figure legend 2D, specify that the last set of values (i.e. 4.2 +/- 0.3 (n=122)) corresponds to the rescue.

Corrected

5) Figure legend 3, correct the grammar in the heading.

The grammar was corrected

© 2021. Published by The Company of Biologists under the terms of the Creative Commons Attribution License (https://creativecommons.org/licenses/by/4.0/).

Second decision letter

MS ID#: JOCES/2020/258364

MS TITLE: INPP5E controls ciliary localization of phospholipids and odor response in olfactory sensory neurons

AUTHORS: Kirill Ukhanov, Cedric Uytingco, Warren Green, Lian Zhang, Stephane Schurmans, and Jeffrey R Martens
ARTICLE TYPE: Research Article

I am happy to tell you that your manuscript has been accepted for publication in Journal of Cell Science, pending standard ethics checks. Thank you for submitting this interesting manuscript.

© 2021. Published by The Company of Biologists under the terms of the Creative Commons Attribution License (https://creativecommons.org/licenses/by/4.0/).
